# Fibroblast Activation Protein Alpha-Targeted Nanoparticles for Tumor Microenvironment Remodeling and Antitumor Therapy in Triple-Negative Breast Cancer

**DOI:** 10.34133/bmr.0347

**Published:** 2026-04-08

**Authors:** Ana Lameirinhas, Paula Díez, Francisco J. Hicke, Anna Ágreda-Roca, Sandra Torres-Ruiz, Paloma Sánchez-Serrano, Marta Tapia, Ana Lluch, Begoña Bermejo, Juan Miguel Cejalvo, Ramón Martínez-Máñez, Pilar Eroles, Iris Garrido-Cano

**Affiliations:** ^1^ Biomedical Research Institute INCLIVA, Breast Cancer Biology Research Group, Valencia, Spain.; ^2^ Instituto Interuniversitario de Investigación de Reconocimiento Molecular y Desarrollo Tecnológico (IDM), Universitat Politècnica de València, Universitat de València, Valencia, Spain.; ^3^ Unidad Mixta de Investigación en Nanomedicina y Sensores. Universitat Politècnica de València, Instituto de Investigación Sanitaria La Fe (IIS La Fe), Valencia, Spain.; ^4^ CIBER de Bioingeniería, Biomateriales y Nanomedicina (CIBER-BBN), Instituto de Salud Carlos III, Valencia, Spain.; ^5^ Clinical Oncology Department, Hospital Clínico Universitario de Valencia, Valencia, Spain.; ^6^ CIBER de Oncología (CIBERONC), Instituto de Salud Carlos III, Valencia, Spain.; ^7^ Department of Medicine, University of Valencia, Valencia, Spain.; ^8^ Unidad Mixta UPV-CIPF de Investigación en Mecanismos de Enfermedades y Nanomedicina, Universitat Politècnica de València, Centro de Investigación Príncipe Felipe, Valencia, Spain.; ^9^ Department of Physiology, University of Valencia, Valencia, Spain.

## Abstract

Triple-negative breast cancer (TNBC) remains a highly aggressive subtype with limited treatment options and poor prognosis. While most therapies target tumor cells, the tumor microenvironment (TME), particularly cancer-associated fibroblasts (CAFs), plays a key role in tumor progression, therapy resistance, and immune suppression.

We developed NP-FAP-DOX, a mesoporous silica nanoparticle-based nanodevice loaded with doxorubicin and functionalized with a fibroblast activation protein alpha (FAP-α) ligand peptide for selective binding to FAP-α-positive CAFs. FAP-α targeting ability and cytotoxic effect were assessed in TNBC cells, patient-derived CAFs, and patient-derived organoids (PDOs). In vivo efficacy was evaluated in the murine orthotopic TNBC 4T1 allograft model, assessing tumor growth inhibition, toxicity, CAFs depletion, extracellular matrix degradation, apoptosis induction, and TME-associated cellular and stromal changes. NP-FAP-DOX exhibited controlled drug release, selective binding to FAP-α, and cytotoxicity in TNBC cells, patient-derived CAFs, and PDOs. In vivo, NP-FAP-DOX reduced tumor growth, depleted CAFs, degraded the extracellular matrix, induced apoptosis, increased lymphocyte infiltration, and decreased M2-like macrophages. Compared with free doxorubicin, NP-FAP-DOX enhanced therapeutic efficacy while reducing cardiotoxicity and systemic side effects. These findings highlight NP-FAP-DOX as a promising nanomedicine strategy for TNBC, integrating tumor inhibition, TME remodeling, and immune activation, with strong potential for clinical translation.

## Introduction

Despite recent advances in breast cancer (BC) therapies, it remains the leading cause of cancer-related deaths in women, with relapse rates continuing to pose a significant challenge despite recent therapeutic advancements [1]. Among BC subtypes, triple-negative BC (TNBC) tumors exhibit an aggressive behavior and are associated with a poor prognosis due to their high metastatic potential compared to other subtypes [2]. In TNBC, chemotherapy remains the standard treatment, often resulting in limited efficacy and a shorter overall survival compared to other BC subtypes [3]. This underscores the urgent need for innovative approaches to improve therapeutic outcomes in this high-risk population.

While most BC therapies currently used in clinical practice focus on targeting tumor cells, the tumor microenvironment (TME), specifically cancer-associated fibroblasts (CAFs), plays a crucial role in tumor progression, tumor immunity modulation, and therapy resistance [4]. In TNBC, the dynamic interplay between cancer cells and the TME not only drives tumor growth but also significantly influences the response to therapies [5]. Indeed, the application of immunotherapy in patients with advanced TNBC has resulted in significantly longer progression-free survival than chemotherapy alone, highlighting the importance of targeting the TME in this context [6].

CAFs, the most abundant stromal cell type in BC [7], are actively involved in tumor-promoting processes such as cancer cell proliferation, invasion [8], drug resistance [9], extracellular matrix (ECM) remodeling [10], suppression of antitumor immunity [11], and promotion of angiogenesis [12]. These features contribute to therapy resistance, partly by altering ECM properties and limiting drug penetration. Importantly, CAF depletion has been shown to inhibit tumor growth and metastasis in BC, supporting their role as a promising therapeutic target [13].

Among CAF markers, fibroblast activation protein alpha (FAP-α) has emerged as a particularly attractive target. FAP-α is overexpressed in activated stromal fibroblasts across multiple tumor types, including BC, and is associated with enhanced tumor progression and metastasis [14]. In TNBC, CAF enrichment, activation, and FAP-α expression levels are significantly higher than in other BC subtypes [15]. Moreover, FAP-α-positive CAFs promote invasion, metastasis, and resistance to immunotherapies in BC [15,16], reinforcing the rationale for FAP-α-targeted therapeutic strategies.

In parallel, nanoparticles (NPs)-based drug delivery systems have emerged in cancer therapy [17]. NPs offer unique characteristics, such as improved drug stability and solubility, controlled release, and selective tumor targeting, thereby reducing off-target effects and enhancing therapeutic efficacy [18]. Among them, mesoporous silica NPs (MSNs) stand out due to their excellent biocompatibility, structural stability, and favorable clearance and excretion profiles [19]. In addition, MSNs exhibit a high drug-loading capacity and functionalization versatility, allowing for specific and efficient controlled cargo release at the target site in response to specific external or internal stimuli, minimizing off-target toxicity [20,21]. Moreover, surface functionalization with (bio)organic groups enhances colloidal stability, biocompatibility, and cellular internalization [22,23]. Besides, specific ligands such as antibodies, peptides, or aptamers can be included on the MSN surface to achieve active and cell-specific targeting [24]. Thus, these properties have supported the use of MSNs in BC drug delivery and immunomodulatory applications [25–27].

On the other hand, doxorubicin is a widely used chemotherapeutic agent in BC treatment due to its potent cytotoxic activity. However, its clinical application is severely limited by a narrow therapeutic window and dose-dependent systemic toxicity, including cardiotoxicity and damage to other organs [28]. Nanotechnology-based formulations of doxorubicin, such as liposomal doxorubicin, have demonstrated that improving drug delivery can significantly reduce adverse effects while maintaining antitumor efficacy, supporting the use of NP-mediated strategies to enhance the therapeutic index of this drug [29].

Based on the rationale detailed above, we report herein the preparation of NP-FAP-DOX, an MSN-based nanodevice loaded with doxorubicin and functionalized with a FAP-α targeting peptide, designed to selectively deliver the drug to both tumor cells and CAFs in TNBC. This dual-targeting approach aims to remodel the TME, enhance intratumoural drug penetration, and ultimately improve therapeutic outcomes in TNBC.

## Materials and Methods

### Commercial cell lines

All cell lines were obtained from the American Type Culture Collection (ATCC, VA, USA). Cells were maintained in Dulbecco’s Modified Eagle Medium/Nutrient Mixture F-12 (DMEM/F-12, Biowest, France) supplemented with 10% heat-inactivated fetal bovine serum (FBS, Gibco, NY, USA), 1% L-glutamine 100X 200 mM (Biowest), and 1% sterile filtered penicillin/streptomycin (P/S, Biowest) at 37 °C and 5% CO_2_ in a humidified chamber. All cell lines were routinely tested and confirmed to be free of mycoplasma contamination. All experiments were performed with cell lines in passages lower than 30.

### Cancer-associated fibroblasts

CAFs were obtained from TNBC tumor biopsies at the Hospital Clínico Universitario de Valencia (Spain). Primary tumor biopsies were taken before any patients’ treatment and were analyzed by an expert pathologist to ensure > 20% tumor infiltration and to confirm the BC subtype by immunohistochemical analysis.

The biopsy was washed with 10% P/S diluted in phosphate-buffered saline (PBS) for 10 min at room temperature, following mechanical disaggregation. The sample was digested with 300 U/ml of collagenase (Gibco) and 100 U/ml of hyaluronidase (Sigma-Aldrich, MO, USA) and resuspended in Advanced-DMEM (Biowest) with 1% P/S for 1 h at 37 °C with shaking. After tissue digestion, cells were centrifuged at 300 × *g* for 5 min at room temperature. Cell pellet was incubated for 3 min at room temperature with ammonia–chloride–potassium (ACK) lysis buffer (Gibco). Supplemented culture medium was added to inactivate the reaction, and the sample was centrifuged at 300 × *g* for 5 min at room temperature. Cells were resuspended in supplemented medium and filtered using a 100-μm cell strainer (PluriSelect, CA, USA) and centrifuged at 300 × *g* for 5 min at room temperature. Cells were resuspended in DMEM/F-12 supplemented with 10% FBS, 1% L-glutamine, and 1% P/S and maintained at 37 °C and 5% CO_2_ in a humidified chamber. CAFs were allowed to grow and maintained as described for the commercial cell lines. All experiments were performed with CAFs between passages 3 and 10.

Patient-derived CAF cultures were routinely characterized every 2 passages to confirm phenotype and exclude contamination by other stromal or epithelial cells. For that, cells were resuspended in eBioscience Flow Cytometry staining buffer (FACS buffer, Invitrogen, USA) and incubated with an antibody cocktail containing anti-CD326-Brilliant Violet 421 (324220, BioLegend, USA), anti-CD31-APC-Vio 770 (130-110-672, Miltenyi Biotec, Germany), anti-CD45-Vio Bright R720 (130-127-376, Miltenyi Biotec), and anti-CD90-APC (130-114-861, Miltenyi Biotec) for 30 min at room temperature. Flow cytometry analysis was performed on the LSR FORTESSA X-20 analyzer (BD Biosciences, USA), and data analysis was performed using FlowJo software (version 10.0.7r2, LLC, USA).

### Patient-derived organoids

Primary tumor biopsies taken before performing patients’ treatment were implanted in the mammary fat pad of 6- to 8-week-old non-obese diabetic/severe combined immunodeficiency (NOD/SCID) female mice purchased from Charles River Laboratories (MA, USA) and maintained at the Animal Facilities from the Universitat de Valencia (Valencia, Spain). Tumors were allowed to grow until they reached the institutional euthanasia criteria for tumor size, and they were processed following the same protocol described for obtaining CAFs.

The tumor was washed, followed by mechanical and chemical digestion. The reaction was stopped using patient-derived organoid (PDO) culture medium (Table S1), and the sample was centrifuged at 300 × *g* for 5 min at room temperature. Cells were then resuspended in PDO culture medium and filtered through a 100-μm cell strainer before centrifuging at 300 × *g* for 5 min. A total of 200,000 cells were seeded in a dome of 200 μl of 75% Matrigel Growth Factor Reduced Basement (Corning, NY, USA) in a tissue-treated 6-well plate. After incubating for 15 min at 37 °C, 5 ml of PDOs’ culture medium were added. For PDO maintenance, culture medium was changed twice a week, and PDOs were passed every 7 to 14 d. Before in vitro experiments, the PDOs’ estrogen receptor (ER), progesterone receptor (PR), human epidermal growth factor receptor 2 (HER2), and Ki67 protein expression were evaluated by immunocytochemistry to ensure the maintenance of the characteristics of the tumor of origin.

### Gene expression studies

RNA extraction was carried out using the Trizol reagent method. RNA (1,000 ng) was reverse transcribed using the High-Capacity cDNA Reverse Transcription Kit (Applied Biosystems, MA, USA), according to the manufacturer’s instructions. Real-time quantitative polymerase chain reaction (RT-qPCR) was performed in a QuantStudio 5 Real-Time PCR System (Thermo Fisher Scientific, MA, USA) using 2 μl of resulting complementary DNA (cDNA), 5 μl of TaqMan Universal Master Mix (Applied Biosystems), 0.5 μl of TaqMan 20× assays (human *FAP-α*: Hs00990791_m1, human *GAPDH*: Hs03929097_g1, mouse *Fap-α*: Mm01329177_m1, mouse *Gapdh*: Mm99999915_g1, mouse *Mki67*: Mm01278617_m1, mouse *Myh7*: Mm00600555_m1; Applied Biosystems), and 2.5 μl of RNase-free water, according to the manufacturer’s instructions. *FAP-α* and *MKI67* transcript levels were evaluated using the 2^−ΔCt^ method, and *GAPDH* was used as a reference gene to normalize results. All the samples were run in triplicate.

### Western blot

Total protein was extracted from cells using Pierce RIPA buffer (Thermo Fisher Scientific) supplemented with protease and phosphatase inhibitor cocktail (Thermo Fisher Scientific), and sonication was performed by the Sonics Vibra Cell VC 505 (Sonics & Materials, CT, USA) (10 s at 40% pulse). Protein concentration was determined using the Pierce BCA Protein Assay Kit (Thermo Fisher Scientific), according to the manufacturer’s procedures. Total protein (30 μg) was separated by 10% polyacrylamide gel by sodium dodecyl sulfate–polyacrylamide gel electrophoresis and transferred into an immunoblot nitrocellulose membrane (Bio-Rad, CA, USA) in a 25 mM Tris-base/glycine buffer using a Trans-Blot Turbo Transfer system (Bio-Rad). Membranes were blocked with 5% bovine serum albumin (BSA) in Tris-buffered saline/0.1% Tween 20 (TBS/T) for 1 h at room temperature. Then, membranes were incubated overnight at 4 °C with specific primary antibodies for FAP-α (ab53066, dilution 1:500, Abcam, UK) and loading control GAPDH (#MA5-15738, dilution 1:100, Thermo Fisher Scientific). After incubation, membranes were washed in TBS/T and incubated with the secondary antibody (anti-rabbit #7074 or anti-mouse #7076, dilution 1:2,000, Cell Signaling, MA, USA) for 1 h at room temperature. After washing with TBS/T, proteins were detected by chemiluminescence using Pierce ECL Western Blotting Substrate (Thermo Fisher Scientific) according to the manufacturer’s instructions using the ImageQuant LAS 4000 system (GE Healthcare, IL, USA).

### NPs’ synthesis

To synthesize NPs, 1 g of the structure-directing agent N-cetyltrimethylammonium bromide (Sigma-Aldrich) was dissolved in 480 ml of deionized water, and the solution was magnetically stirred. Then, 3.5 ml of sodium hydroxide (NaOH, Sigma-Aldrich) 2 M was added to increase the pH, followed by a temperature adjustment to 80 °C. After that, 5 ml of tetraethyl orthosilicate (TEOS, 2.57 × 10^−2^ mol, Sigma-Aldrich) was added dropwise to the solution, and the mixture was stirred for 2 h, allowing the formation of a white precipitate (as-synthesized NPs). The precipitate was isolated and collected by centrifugation, washed with deionized water, and dried at 70 °C. Finally, the solid was calcined for 5 h at 550 °C to remove the template phase, obtaining the final MSNs.

To prepare the NP-CTR-DOX and NP-FAP-DOX, 100 mg of NP was dispersed in 6 ml of anhydrous acetonitrile (ACN) and reacted with 98 ml of (3-mercaptopropyl)-trimethoxy silane for 5.5 h. Then, 112 mg of 4,4′-dipyridyl disulfide was added to the mixture, and the reaction was magnetically stirred for 24 h. The thiolated solid was washed with ACN, ethanol, and PBS, and stirred for 24 h after adding 98 mg of doxorubicin in 8 ml of PBS. The solid was then isolated by centrifugation and resuspended in 8 ml of ACN, and 195 mg of SH-PEG_12_-COOH (polyethylene glycol [PEG]) was added, keeping the mixture stirred overnight to form the molecular gate. To attach the peptide, first, the NPs were washed with ACN, ethanol, and PBS, and after that, they were resuspended in 8 ml of PBS, pH 6, where 50 mg of *N*-ethyl-*N*′-(3-dimethylaminopropyl)carbodiimide hydrochloride and 50 mg of *N*-hydroxysuccinimide were added to activate the carboxylic group of the molecular gate. The mixture was stirred for 2 h. Then, the activated NPs were collected by centrifugation and resuspended in 8 ml of water at pH 7. To this suspension, 100 mg of a control peptide (CTR peptide sequence: Gly-Gly-Gly-Gly-Gly-Gly-Gly-Ala-Thr-Lys-Asp-Ala-Thr-Gly-Asp-Lys-Thr-Ala) or a FAP-α ligand peptide with a high affinity for FAP-α (FAP-α ligand peptide sequence: Gly-Gly-Gly-Gly-Gly-Gly-Gly-Ala-Thr-Lys-Asp-Ala-Thr-Gly-Pro-Ala-Lys-Thr-Ala) were added, and the mixture was stirred overnight. The final solids (NP-CTR-DOX and NP-FAP-DOX) were washed with water and dried at 37 °C.

The control nanodevices without doxorubicin (NP-CTR and NP-FAP) were prepared following the same procedure without the drug-loading process.

### NPs’ characterization

Powder x-ray diffraction (PXRD) patterns were obtained by a diffractometer using Cu-Kα radiation (D8 Advance, Philips Amsterdam, Netherlands). High-resolution transmission electron microscopy (HR-TEM) images were taken using a JEOL TEM-2100F electron microscope (JEOL Europe SAS, France). Elemental mapping was conducted using scanning transmission electron microscopy coupled with electronic energy-dispersive x-ray spectroscopy (STEM-EDX) using a JEM 2100F instrument. Thermogravimetric analysis (TGA) was performed with a TA Instruments SDTQ600 apparatus (Mettler Toledo, Inc., Switzerland) in an oxidizing atmosphere (air, 80 ml min^−1^). The loss of weight was registered within a dynamic step with a heating program consisting of a heating rate of 10 °C/min from 25 to 100 °C, an isothermal heating step at 100 °C for 60 min, a heating rate of 10 °C/min from 100 °C to 1,000 °C, and an isothermal heating step at this final temperature for 30 min. Fourier Transform infrared spectroscopy (FTIR) measurements were performed in a Tensor 27 instrument (Bruker, Germany). Dynamic light scattering (DLS) and ζ potential evaluations were carried out using a Zetasizer Nano ZS (Malvern Panalytical, UK). Ultraviolet–visible measurements were performed with a JASCO V-650 spectrophotometer (Jasco, MD, USA). Fluorescence measurements were carried out in a JASCO FP-8500 spectrophotometer.

### Control release studies

For in vitro cargo-controlled release studies, 6 mg of NP-FAP-DOX was suspended in 1 ml of 10 mM PBS, pH 7.5, and separated into 6 fractions. Then, 1 ml of PBS or glutathione (GSH) was added, and the suspensions were incubated for 3 h on a shaker (PCMT Thermo-shaker HC24N, Grant Instruments, UK) at 37 °C. At successive times (5, 15, 30, 60, 180, and 210 min), the mixtures were centrifuged (5 min, 12,500 rpm) to remove solids, and the fluorescence emission of doxorubicin released to the medium was measured (*λ*_exc_ = 470 nm, *λ*_em_ = 555 nm).

To determine the total amount of doxorubicin contained in the NPs, forced release assays were performed. For that purpose, 1 mg of doxorubicin-containing NPs was suspended in 1 ml of dimethyl sulfoxide (DMSO) and stirred for 24 h in the dark. The suspensions were then centrifuged, and the absorbance of the supernatant was measured (*λ*_abs_ = 495 nm) in a JASCO FP-8300 spectrophotometer. Doxorubicin concentration was calculated by Lambert–Beer's law (A=ε·c·l), where *A* is absorbance, c is the molar concentration (M), l is the optical length path (cm), and ε is the molar absorption coefficient of doxorubicin (9,250 M^−1^ cm^−1^).

### Hemolysis assay

Blood from BALB/C mice was collected in ethylenediaminetetraacetic acid (EDTA) tubes and red blood cells were isolated after centrifuging at 500 × *g* for 5 min. Cells were washed 3 times with PBS and diluted in the same buffer (1:50). Then, 180 μl of red blood cell suspension was incubated at 37 °C with 20 μl of NPs (NP-CTR, NP-FAP, NP-CTR-DOX, and NP-FAP-DOX) at different concentrations (final concentrations of 10, 25, 50, 100, 250, 500, 750, and 1,000 μg/ml). PBS and 1% Triton X-100 in PBS were used as negative and positive controls, respectively. After 1 h, samples were centrifuged at 500 × *g* for 5 min, and the hemoglobin release was determined by measuring the supernatant absorbance at 540 nm in a microplate reader Spectra Max Plus (Thermo Fisher Scientific). The percentage of hemolysis was calculated as [(sample absorbance − negative control) / (positive control − negative control) × 100].

### Cellular uptake and targeting efficacy

Cell lines (40,000 cells/well) and CAFs (15,000 cells/well) were seeded in an 8-well chambered coverslip (Ibidi GmbH, Germany) and incubated for 3 h with 25 μg/ml of NP-CTR-DOX and NP-FAP-DOX. The nuclei were stained with 4',6-diamidino-2-phenylindole (DAPI, 5 μg/ml, Merck, Germany). The doxorubicin and DAPI fluorescence were analyzed by fluorescence microscopy using the Leica DMi8 inverted fluorescence microscope (Leica Microsystems, Germany) with a PE4000 LED light source and DFC9000GT camera at 20× magnification at the Central Medicine Research Unit of Universitat de Valencia (UCIM-UV). A total of 15 pictures per well were taken with an excitation of 365 and 550 nm, and an emission of 435 to 485 nm and 615 nm for DAPI and doxorubicin, respectively. Doxorubicin mean fluorescence intensity per cell was quantified using Image J Software (version 1.51h, NIH). Regions of interest (ROIs) corresponding to individual cells were generated, and the mean fluorescence intensity of doxorubicin per cell was measured. For each condition, a minimum of 200 cells were analyzed, and the fluorescence intensity of NP-FAP-DOX was normalized for NP-CTR-DOX.

BC PDOs (3,000 aggregates/dome) were seeded in a dome of 30 μl of 75% Matrigel Growth Factor Reduced Basement (Corning) in an 8-well chambered coverslip (Ibidi GmbH) and incubated with 25 μg/ml of NP-CTR-DOX and NP-FAP-DOX for 24 h. The nuclei were stained with Hoechst 33342 (Invitrogen, MA, USA) for 4 h. NP penetration was analyzed by confocal microscopy using the Leica TCS SP8 X White Light Laser Confocal Microscope (Leica Microsystems) with an HC PL APO CS2 20x/0.75 IMM objective at the UCIM-UV. Five pictures per well were taken in sequential order with an excitation of 405 and 546 nm, and an emission of 380 to 547 nm and 584 to 637 nm for Hoechst and doxorubicin, respectively. ROIs were defined for each organoid, and the mean doxorubicin fluorescence intensity per organoid was calculated using Image J Software (version 1.51h, NIH). For each condition, a minimum of 30 organoids were analyzed, and the fluorescence intensity of NP-FAP-DOX was normalized to NP-CTR-DOX.

### Cellular cytotoxicity

Cells (8,000 cells/well) and CAFs (3,000 cells/well) were plated into 96-well plates and treated with NP-CTR, NP-FAP, or NP-FAP-DOX at different concentrations (0, 1, 5, 10, 25, and 50 μg/ml). After 72 h, cell viability was evaluated by using the Colorimetric Cell Viability Kit II (WST-1, Deltaclon, Spain) according to the manufacturer’s instructions. Spectrophotometric analysis was measured at 450 nm (reference wavelength: 650 nm) in a microplate reader Spectra Max Plus (Thermo Fisher Scientific).

BC PDOs (10,000 cells/well) were seeded in 96-well plates as single cells and treated with NP-FAP or NP-FAP-DOX at different concentrations (0, 1, 5, 10, 25, and 50 μg/ml). After 72 h, PDO viability was determined by using the CellTiter-Glo 3D Cell Viability Assay (Promega, WI, USA), according to the manufacturer’s instructions. Luminescence was measured in the luminometer Promega GloMax Plate Reader (Promega).

### Apoptosis analysis

Cells (8,000 cells/well) and CAFs (3,000 cells/well) were plated into 96-well plates and treated with NP-CTR, NP-FAP, or NP-FAP-DOX at different concentrations (0, 1, 5, 10, 25, and 50 μg/ml). Untreated cells were included as a control. After treatment, cells were stained with FITC annexin V (Immunostep, Salamanca, Spain) as recommended by the manufacturer. Samples were acquired using BD LSRFortessa (BD Biosciences, Franklin Lakes, NJ, USA), and data were analyzed by FlowJo software (version 10.0.7r2).

### Clonogenic assay

Cells were treated with CDDP and PtL2 for 3 and 24 h (10 and/or 20 μM). Then, cells were cultured in a 6-well plate at a very low concentration (2 × 10^3^ cells per well) and incubated for 10 d.

Cells were treated with NP-CTR, NP-FAP, or NP-FAP-DOX at different concentrations (0, 1, 2.5, and 5 μg/ml) for 3 h. Then, cells were washed and cultured in a 6-well plate at a very low concentration (500 cells per well). After 10 d, cells were fixed with 4% paraformaldehyde for 15 min, stained with 0.5% crystal violet for 15 min, washed with distilled water, and air-dried. The clone formation number was counted with Image J Software (version 1.51h, NIH).

### In vivo studies

The in vivo experiments were performed in 6- to 8-week-old BALB/C female mice purchased from Charles River Laboratories and maintained at the animal facilities of UCIM-UV. To generate the in vivo tumor model, 250,000 4T1 cells were injected into the mammary fat pad of BALB/C mice, and the tumors were allowed to grow. Animals were housed 6 per cage under conditions compliant with EU Directive 63/2010, with environmental enrichment, and were monitored following Morton and Griffiths’ (1985) protocol to assess welfare. Humane endpoints were established: animals reaching a welfare score of 9 points or tumor volume exceeding 1 cm^3^ were euthanized to minimize pain, suffering, and distress. Once tumors were palpable, mice were randomly divided into groups of 12 animals and treated separately with PBS or 50 mg/kg of NPs (NP-CTR-DOX and NP-FAP-DOX) 4 times per week for 15 d. Additionally, another group of 12 animals was treated with 3.75 mg/kg of free doxorubicin for 8 d. All treatments were administered intravenously (via tail) and prepared in free-serum DMEM-F12 medium every day before administration. The NP suspension was slightly sonicated for 30 min to avoid NP aggregates. Tumors were measured 4 times per week, and tumor volume was calculated using the formula (*D* × *d*^2^)/2, based on the longest (*D*) and the shortest (*d*) tumor diameters. Body weight was registered 4 times per week, and mice were observed continuously for normal health conditions and behavior. Mice were sacrificed 3 d after the last treatment or when they met the institutional euthanasia criteria for tumor size or overall health condition.

After mice were sacrificed, tumors, hearts, liver, spleen, and kidneys were collected. For histological analysis, tissues were fixed in 4% paraformaldehyde (VWR BDH Chemicals, USA) and embedded in paraffin. Hematoxylin and eosin H&E) staining was performed in 4-μm-thick sections of formalin-fixed, paraffin-embedded (FFPE) tissue samples of the heart to evaluate cardiotoxicity. Representative photographs were taken in a Leica DM 2500 LED microscope (Leica Microsystems) with a digital camera MC170 HD at 4× and 20× magnification at the UCIM-UV.

### Doxorubicin penetration

FFPE tumor 4-μm-thick sections were deparaffinized and rehydrated. Samples were incubated for 15 min at room temperature with 0.25% Triton X-100 in PBS for cell permeabilization. Nuclear staining was performed by incubating with DAPI (5 μg/ml, Merck) for 10 min at room temperature. Following, the slides were mounted using 50% glycerol in PBS. Doxorubicin intensity was analyzed by confocal microscopy using the Leica TCS SP8 X White Light Laser Confocal Microscope (Leica Microsystems) with an HC PL APO CS2 20×/0.75 IMM objective at the UCIM-UV. Pictures were taken sequentially with an excitation of 405 and 546 nm, and an emission of 410 to 500 nm and 584 to 637 nm for DAPI and doxorubicin, respectively.

### Masson’s trichrome staining

Masson’s trichrome staining was performed to evaluate the ECM content by selectively staining the collagen fibers, muscle, and connective tissue. For that, 4-μm-thick sections of FFPE tumor samples were used. Automated staining was carried out using the Dako Artisan Link Pro (Dako, Denmark) with Masson’s Trichrome Stain Kit (AR173, Dako). Representative photographs were taken in Leica DM 2500 LED microscope (Leica Microsystems) with a digital camera MC170 HD at 20× magnification at the UCIM-UV.

### Immunofluorescence

FFPE tumor sections of 4 μm thickness were pretreated in the DAKO PT Link Pretreatment module (#PT100/PT101, Dako), and antigen retrieval was performed with Dako High Retrieval Solution (EDTA buffer) for 30 min. Samples were incubated for 15 min at room temperature with 0.25% Triton X-100 in PBS, blocked with 5% BSA in PBS for 1 h at room temperature, and incubated with alpha-smooth muscle actin (α-SMA)-Alexa Fluor 488 antibody (ab184675, dilution 1:100, Abcam) or cleaved caspase-3 (#9661, dilution 1:200, Cell Signaling) diluted in 2.5% BSA in 1× PBS, overnight at 4 °C. On the following day, samples stained with cleaved caspase-3 were incubated with anti-rabbit-Alexa Fluor 488 secondary antibody (#A11034, dilution 1:500, Invitrogen) for 1 h at room temperature. Samples were stained with DAPI (5 μg/ml, Merck) for 10 min at room temperature, and the slides were mounted using 50% glycerol in PBS. Immunofluorescence staining was analyzed by confocal microscopy using the Leica TCS SP8 X White Light Laser Confocal Microscope (Leica Microsystems) with an HC PL APO CS2 20×/0.75 IMM objective at the UCIM-UV. Pictures were taken in sequential order with an excitation of 405 nm and an emission of 410 to 500 nm for DAPI, or with an excitation of 448 nm and an emission of 505 to 544 nm for α-SMA or cleaved caspase-3.

### Second-harmonic generation microscopy

Second-harmonic generation (SHG) microscopy was performed to visualize and assess the organization of fibrillar collagen within the tumor ECM. FFPE tumor sections (5 μm thick) were first deparaffinized, rehydrated through graded ethanol, and mounted using an aqueous mounting medium without staining to preserve intrinsic SHG signals. SHG imaging was conducted using a multiphoton laser scanning microscope (Leica TCS-4Pi) equipped with a 25× water-immersion objective. Images were acquired with identical acquisition settings across all samples to allow for comparison (excitation: 850 nm, emission: 418 to 432 nm).

### Characterization of TME composition by flow cytometry

Tumor samples were disaggregated using scalpels and digested with a digestion solution (0.015 g collagenase and 0.001 g hyaluronidase in 10 ml PBS) for 45 min at 37 °C in agitation. Tissue was dissociated with a mechanical pistol, and RNase-free DNase I (Qiagen, Germany) was added. After 10 more minutes of incubation, the digestion was stopped by adding 10% FBS in PBS, and samples were centrifuged at 800 rpm for 6 min at 4 °C. Red blood cells were eliminated by using ACK lysis buffer (Gibco). After 3 min of incubation, 10% FBS in PBS was added, and the mixture was filtered through 100-μm strainers. The samples were centrifuged at 800 rpm for 4 min at 4 °C, and the pellet was resuspended in PBS for further staining. Samples were stained with Zombie Aqua (BioLegend) diluted 1:4 in DMSO for 30 min in the dark at room temperature. For surface staining, cells were resuspended in FACS buffer (Invitrogen) and incubated with the surface antibodies (Table S2) for 30 min in the dark at 4 °C. After incubation, samples were fixed with 4% paraformaldehyde for 10 min at room temperature, washed with PBS, and resuspended in FACS buffer. Flow cytometry analysis was performed on the LSR FORTESSA X-20 analyzer (BD Biosciences, CA, USA), and data analysis was conducted using FlowJo software (version 10.0.7r2).

### Statistical analysis

Statistical analysis was performed using the GraphPad Prism 8.0.1 software (GraphPad Software Inc., La Jolla, MA, USA). Data normality was assessed using the Shapiro–Wilk test. For comparisons between 2 groups, a 2-tailed Student *t* test was applied to normally distributed data, whereas the nonparametric Mann–Whitney *U* test was used when normality was not met. For comparisons involving more than 2 groups, one-way analysis of variance followed by Tukey’s multiple comparisons test was used for parametric data, while the Kruskal–Wallis test followed by Dunn’s multiple comparisons test was applied for nonparametric data. Adjusted *P* values derived from these post hoc analyses are reported in the figures. Data are presented as mean ± standard deviation (SD) or mean ± standard error of the mean (SEM), as indicated in the figure legends. All experiments were performed at least in biological and technical triplicates. *P* values were considered statistically significant when inferior to 0.05. Significance is shown vs. the respective control and depicted as follows: *P < 0.05, **P < 0.01, ***P < 0.001, and ****P < 0.0001.

## Results

### Synthesis and characterization of NP-FAP-DOX

NPs were synthesized using MSNs as the structural scaffold. First, the surface of MSNs was functionalized with thiol groups, followed by doxorubicin loading into the mesoporous network. Then, the pores were blocked with PEG conjugated via dithiol bonds to the NP’s surface, thus obtaining a redox-sensitive molecular gate (S-S-PEG-COOH). Finally, the FAP-α ligand peptide, designed to ensure the selective recognition of the therapeutic target FAP-α, was conjugated to the PEG-COOH terminal through an amide bond, obtaining the final nanodevice NP-FAP-DOX (Fig. S1A). This design allows the NPs to target FAP-α and release their cargo upon exposure to high GSH concentrations typically found in the intracellular environment. Under these conditions, the dithiol bonds are reduced, triggering delivery of the cargo (Fig. S1B). In addition, control NPs coated with a peptide lacking specificity for FAP-α (NP-CTR-DOX) and unloaded counterparts (NP-FAP and NP-CTR) were also synthesized for comparative studies.

After the synthesis of the NPs, their structure and functionalization were studied by standard methods. Structural characterization was assessed by HR-TEM, which confirmed that the MSN scaffold showed an ordered mesoporous structure and spherical morphology with a diameter of 102 ± 22 nm (Fig. S2A and B). The mesoporous hexagonal network was further demonstrated by the PXRD pattern showing a characteristic MCM-41 type Bragg peak at 2.4°, indexed as a (100) plane in the MSN scaffold. This peak remained preserved after functionalization, indicating that the loading and functionalization process did not modify the mesopore structure observed by HR-TEM (Fig. S2C).

**Fig. 1.**
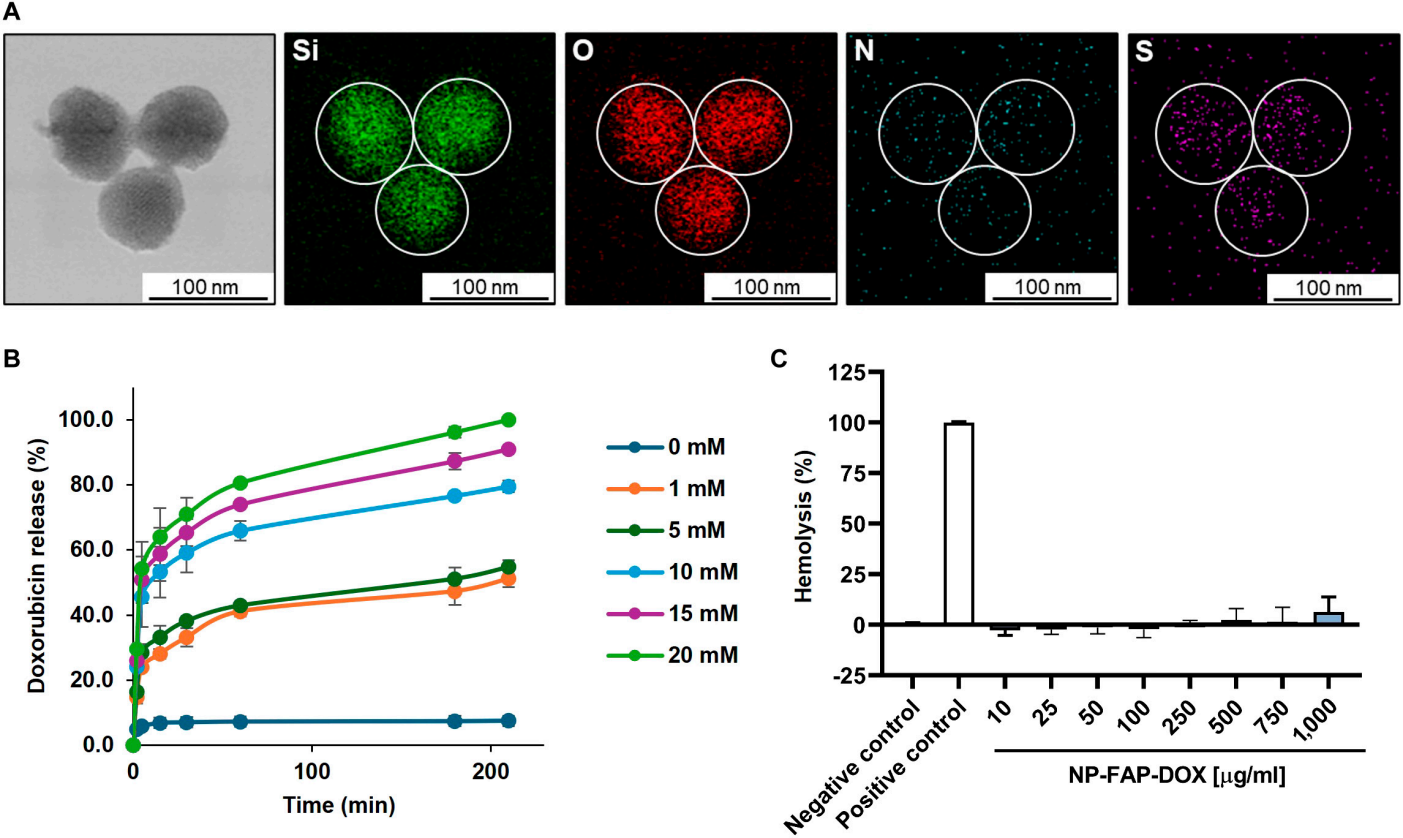
NP-FAP-DOX characterization. (A) Scanning transmission electron microscopy coupled with electronic energy-dispersive x-ray spectroscopy (STEM-EDX) atomic mapping of Si (green), O (red), N (blue), and S (purple) atoms in NP-FAP. Weight (%) composition was calculated in 52.8% of O_2_, 46.7% of Si, and 0.6% of S. (B) Doxorubicin release study from NP-FAP-DOX in the absence or presence of glutathione (GSH) at different concentrations, determined by measuring doxorubicin fluorescence vs. time in phosphate-buffered saline (PBS) (mean ± SD). (C) Hemolytic activity of the NP-FAP-DOX. Red blood cells were incubated with PBS (negative control), 1% Triton X-100 (positive control), or NP-FAP-DOX at different concentrations (mean ± SD).

STEM-EDX showed the presence of Si and O atoms, which confirmed the elemental composition of the MSN scaffold. In addition, the presence of S atoms, assigned to the redox-sensitive molecular gate (S–S bonds), and N atoms, attributed to the FAP*-*α ligand peptide, confirmed the successful functionalization in NP-FAP-DOX (Fig. 1A). Additional characterization was performed using FTIR, which verified the presence of the expected functional groups in NP-FAP and NP-CTR. All the nanomaterials showed the typical bands attributed to stretching vibrations of Si-O-Si bonds at 1,068 and 1,246 cm^−1^ (shoulder), Si-OH groups at 962 cm^−1^, and SiO_4_ tetrahedrons at 810 cm^−1^. Both NP-FAP and NP-CTR spectra showed the amide I absorption band at 1,644 cm^–1^ and stretching vibrations of OH and NH groups at 3,400 cm^–1^, corresponding to the FAP-α ligand and the CTR peptides (Fig. S2D). TGA and elemental analysis allowed to determine content values of 15, 68.7, and 71.5 μg/mg of NPs for PEG, FAP-α ligand peptide, and CTR peptide, respectively (Fig. S2E and Table S3).

Finally, DLS revealed that the hydrodynamic diameter increased from 180 ± 2 nm for MSNs to 247 ± 9 nm for NP-FAP-DOX, with control NPs showing similar sizes (Table S4). Moreover, the ζ potential showed the typical negative surface charge in the mesoporous silica scaffold due to the presence of silanol groups (−34.9 mV), which changed after the functionalization with the PEG (−39.7 mV), loading with doxorubicin (−18.9 mV), and the capping functionalization (−32.8 mV). Control NPs exhibited comparable trends (Table S5).

In addition, the total doxorubicin loading capacity was determined from delivery studies, yielding 82.7 ± 2.5 μg and 81.6 ± 2.2 μg (mean ± SD) per milligram of NP-FAP-DOX and NP-CTR-DOX, respectively.

Once the NP characterization was completed, the capacity of the final NP-FAP-DOX to deliver the loaded doxorubicin in a controlled and redox-responsive manner was further evaluated. In vitro cargo release studies were performed using GSH at concentrations ranging from 1 to 20 mM, simulating the intracellular redox conditions found in cancer cells [30]. All tested GSH concentrations triggered doxorubicin release from NP-FAP-DOX, confirming the redox-sensitive gating mechanism. Notably, doxorubicin release occurred in a GSH concentration-dependent manner, with higher GSH levels inducing a greater extent of cargo release. In contrast, negligible release was observed in PBS, indicating that the NPs effectively retain the drug under nonreducing conditions (Fig. 1B). These results support the ability of NP-FAP-DOX to prevent nonspecific drug release and ensure preferential intracellular delivery within the reductive TME. Besides, hemolysis assays confirmed the biocompatibility of NP-FAP-DOX. Even at high concentrations, the interactions between the NPs and the red blood cells did not induce hemoglobin release, demonstrating the nontoxic profile of NP-FAP-DOX (Fig. 1C). To assess the colloidal stability of NP-FAP-DOX in physiologically relevant conditions, NPs were incubated in 50% FBS in PBS at 37 °C and analyzed by DLS at different time points. The hydrodynamic size distribution remained consistent even after 15 d, with no signs of aggregation (Fig. S2F and Table S6). Importantly, TEM analysis performed after 15 d of incubation confirmed the preservation of NP morphology and mesoporous structure (Fig. S2G). These results confirm that NP-FAP-DOX maintains colloidal stability under serum conditions, supporting its potential use for systemic administration.

### NP-FAP-DOX targeting ability

**Fig. 2.**
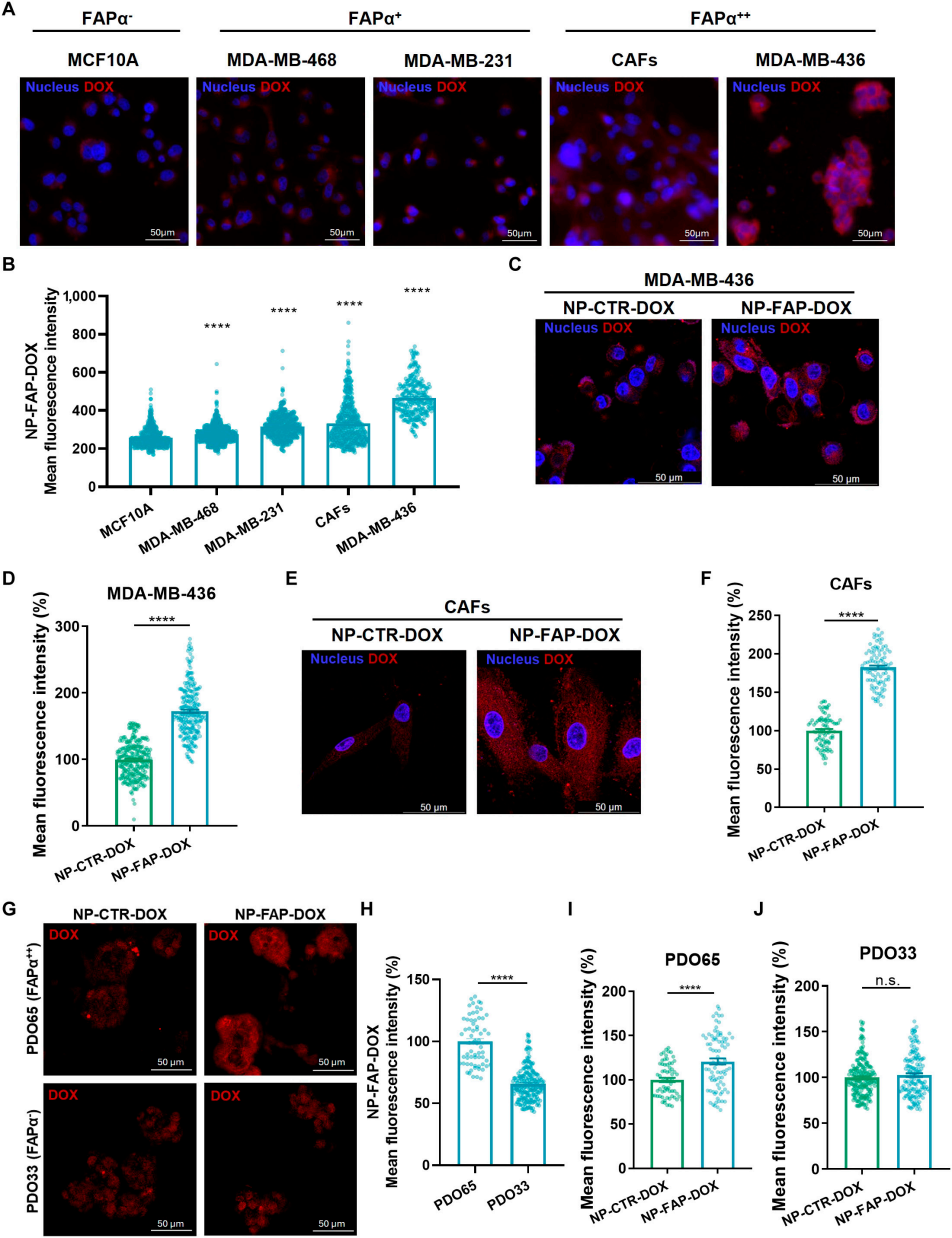
NP-FAP-DOX targeting efficacy. (A and B) MCF-10A, MDA-MB-468, MDA-MB-231, cancer-associated fibroblasts (CAFs), and MDA-MB-436 cells were treated with NP-FAP-DOX (25 μg/ml, 3 h). Intracellular doxorubicin was visualized by fluorescence microscopy. (A) Representative images. (B) Quantification of doxorubicin fluorescence intensity (mean ± SEM). Statistical comparisons were made against MCF-10A. (C to J) Comparative internalization of NP-CTR-DOX vs. NP-FAP-DOX (25 μg/ml, 3 h). Representative images and corresponding quantification of intracellular doxorubicin fluorescence in (C and D) MDA-MB-436 cells, (E and F) CAFs, and (G to J) patient-derived organoids (PDOs). Fluorescence intensity normalized to NP-CTR-DOX (mean *±* SEM). ****P < 0.0001. n.s., nonsignificant.

The targeting capacity of NP-FAP-DOX was assessed in cell lines exhibiting different levels of FAP-α expression: FAP-α-negative MCF-10A, intermediate-expressing MDA-MB-468 and MDA-MB-231, and high-expressing MDA-MB-436 (Fig. S3). Uptake of NP-FAP-DOX correlated with FAP-α expression. MDA-MB-436 showed the highest internalization, whereas uptake in FAP-α-negative cells (MCF-10A) was significantly reduced (approximately 54% of the signal observed in MDA-MB-436) (Fig. 2A and B). MDA-MB-468 and MDA-MB-231 displayed intermediate uptake (approximately 59% and 67% of MDA-MB-436, respectively), but both exhibited significantly higher internalization than MCF-10A cells. These findings indicate that NP-FAP-DOX retains targeting capability even in cells with moderate FAP-α expression, which may reflect the heterogeneous expression patterns observed in tumor tissues.

Cellular uptake and targeting efficacy of NP-FAP-DOX were further compared with control NP loaded with doxorubicin and functionalized with a nonspecific peptide (NP-CTR-DOX). In FAP-α-positive MDA-MB-436, MDA-MB-468, and MDA-MB-231 cells, treatment with NP-FAP-DOX resulted in a significantly higher uptake of doxorubicin compared to control NP-CTR-DOX after 3 h of incubation (approximately 172%, 118%, and 130% higher than the control, respectively) (Fig. 2C and D and Fig. S4A to D). Notably, no differences were observed between NP-FAP-DOX and NP-CTR-DOX in FAP-α-negative MCF-10A cells (Fig. S4E and F).

The targeting efficacy was also confirmed in primary culture of FAP-α-positive CAFs obtained from metastatic TNBC, previously characterized by flow cytometry as CD90-positive and expressing both FAP-α and α-SMA, where NP-FAP-DOX internalization exceeded that of NP-CTR-DOX (approximately 175%) and was higher than in MCF-10A cells (Fig. 2A, B, E, and F and Fig. S5).

Moreover, the tumor penetration and targeting capacities were further evaluated in PDOs from metastatic TNBC with differential FAP-α expression: FAP-α-negative PDO33 and high-expressing PDO65 (Fig. S3). After 3 h of incubation, doxorubicin uptake was higher in PDO65 than in PDO33 (ca. 65% in PDO33 compared to PDO65). When comparing NP-FAP-DOX and NP-CTR-DOX, a significant difference was observed only in the FAP-α-positive PDO65 (approximately 121% vs. control), while no differences were detected in FAP-α-negative PDO33 (Fig. 2G to J).

Collectively, these findings confirm that NP-FAP-DOX selectively targets and is preferentially internalized by FAP-α-expressing TNBC cells, CAFs, and PDOs.

### NP-FAP-DOX cytotoxic efficacy

The cytotoxic effects of NP-FAP-DOX were evaluated in the TNBC cell lines MDA-MB-436, MDA-MB-468, and MDA-MB-231. Cytotoxicity was assessed through WST-1 viability assay, apoptosis detection by Annexin V flow cytometry, and clonogenic assays.

**Fig. 3.**
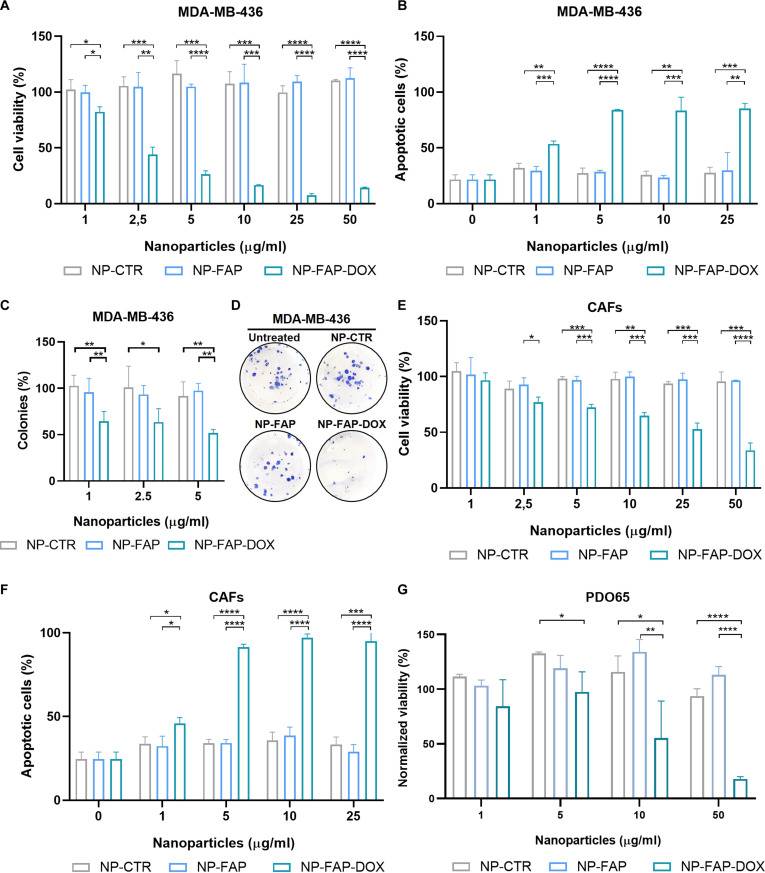
NP-FAP-DOX cytotoxic effect. (A to D) MDA-MB-436 cells, (E and F) CAFs, and (G) PDO65 were treated with NP-CTR, NP-FAP, or NP-FAP-DOX for 72 h. Cell viability was measured using the WST-1 assay for MDA-MB-436 (A) and CAFs (E), and CellTiter-Glo for PDOs (G). Apoptosis was assessed by flow cytometry using Annexin V-FITC staining (B and F). (C and D) Clonogenic assay in MDA-MB-436 cells treated with NP-CTR, NP-FAP, or NP-FAP-DOX for 24 h. (C) Quantification of colony number normalized to the negative control. (D) Representative images of colony formation after treatment with 5 μg/ml. Mean ± SD. *P < 0.05, **P < 0.01, ***P < 0.001, ****P < 0.0001. n.s., nonsignificant.

Control NPs (NP-CTR and NP-FAP) did not affect cell viability, whereas NP-FAP-DOX induced a significant cytotoxic effect in all cell lines, mediated by apoptosis induction. Clonogenic assays confirmed that NP-CTR and NP-FAP did not impact long-term proliferative capacity, while NP-FAP-DOX inhibited colony formation (Fig. 3A to D and Fig. S6). Similar results were obtained in CAFs (Fig. 3E and F) and in PDOs, where NP-FAP-DOX decreased cell viability by up to 80% without significant toxicity from NP-CTR or NP-FAP at the highest concentration tested (Fig. 3G).

These results demonstrate that NP-FAP-DOX exerts potent cytotoxic effects across multiple TNBC models, including CAFs and complex PDO that recapitulate tumor architecture, by inducing apoptosis and markedly reducing clonogenic potential.

### NP-FAP-DOX in vivo antitumor efficacy

**Fig. 4.**
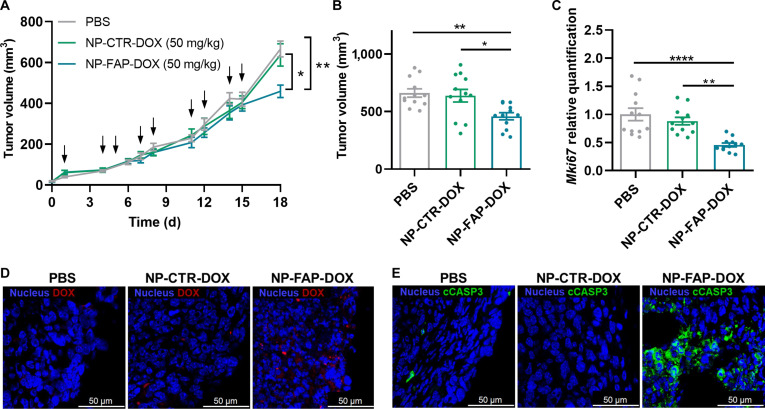
NP-FAP-DOX in vivo antitumoral efficacy. (A) Tumor growth curve of mice following treatment with PBS (vehicle), NP-CTR-DOX, and NP-FAP-DOX (*n* = 12 per group). The arrows indicate the days of treatment administration. (B) Tumor volume at the treatment endpoint (mean ± SEM). (C) *Mki67* expression in tumors at the treatment endpoint determined by real-time quantitative polymerase chain reaction (RT-qPCR) normalized to PBS (mean ± SEM). (D) Representative images of internalized doxorubicin in paraffin-embedded tumor sections at the treatment endpoint. Blue: nucleus, red: doxorubicin. Scale bar: 50 μm. (E) Representative images of immunofluorescence staining of cleaved-caspase 3 (cCASP3) in paraffin-embedded tumor sections at the treatment endpoint. Blue: nucleus, green: cCASP3. Scale bar: 50 μm. *P < 0.05, **P < 0.01, ****P < 0.0001.

The antitumor efficacy of NP-FAP-DOX was evaluated in the murine orthotopic of TNBC 4T1 allograft model. The targeting ability and cytotoxic activity of NP-FAP-DOX were previously confirmed in 4T1 cells (Fig. S7). Tumor-bearing mice were treated with PBS (vehicle), NP-CTR-DOX, or NP-FAP-DOX for 15 d. NP-FAP-DOX treatment significantly inhibited tumor growth compared to both the vehicle (PBS) and the control NPs NP-CTR-DOX (Fig. 4A). By the end of the treatment endpoint, NP-FAP-DOX reduced tumor volume by approximately 30%, whereas no significant differences were observed between the PBS-treated animals and those receiving the control NP (Fig. 4B).

Additionally, the ability of NP-FAP-DOX to suppress tumor cell proliferation was further evidenced by a significant decrease in *Mki67* mRNA expression in NP-FAP-DOX-treated tumors compared to the other groups (Fig. 4C). Consistently, confocal microscopy images showed higher doxorubicin accumulation in the NP-FAP-DOX-treated tumors compared to NP-CTR-DOX, confirming its tumor-targeting capability and efficient drug delivery (Fig. 4D). Furthermore, the antitumor effects of NP-FAP-DOX were confirmed by an increase in the number of apoptotic cells in treated tumors, as shown by cleaved-caspase-3 immunofluorescence staining (Fig. 4E). These findings collectively highlight the potent antitumor activity of NP-FAP-DOX through enhanced tumor targeting, effective drug delivery, and induction of apoptosis.

### Effect of NP-FAP-DOX in modulating TME composition in vivo

**Fig. 5.**
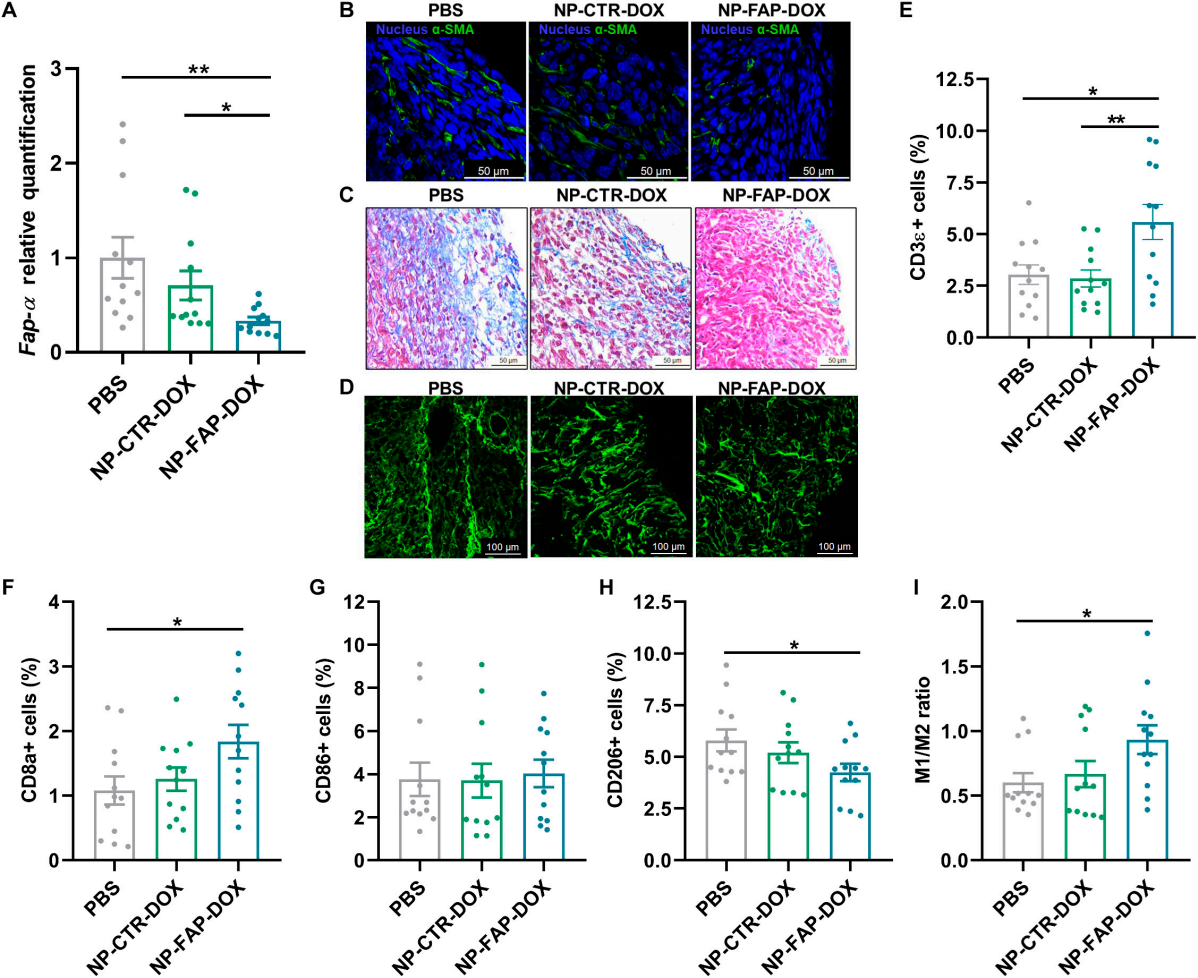
NP-FAP-DOX in vivo TME modulation. (A) *Fap-α* expression in tumors at the treatment endpoint was determined by RT-qPCR normalized to PBS (*n* = 12 per group). Mean *±* SEM. (B) Representative images of immunofluorescence staining of alpha-smooth muscle actin (α-SMA) in paraffin-embedded tumor sections at the treatment endpoint. Blue: nucleus, green: α-SMA. Scale bar: 50 μm. (C) Representative images of Masson’s trichrome staining in paraffin-embedded tumor sections at the treatment endpoint. Blue: collagen, pink: fibrin, red: muscle fibers, black: nucleus. Scale bar: 50 μm. (D) Representative second-harmonic generation (SHG) microscopy images of tumor sections showing fibrillar collagen structure. (E to H) Percentage of (E) T lymphocytes (CD45^+^/CD3ε^+^), (F) cytotoxic T cells (CD45^+^/CD3ε^+^/CD8a^+^), (G) M1-like macrophages (CD45^+^/F4/80^+^/CD86^+^), and (H) M2-like macrophages (CD45^+^/F4/80^+^/CD206^+^) in xenograft tumors analyzed by flow cytometry at the treatment endpoint (*n* = 12 per group). (I) Ratios of M1 to M2 (M1/M2) were calculated as the percentage of M1-like macrophages divided by the percentage of M2-like macrophages on xenograft tumors analyzed by flow cytometry at the treatment endpoint (*n* = 12 per group). Mean *±* SEM. *P < 0.05, **P < 0.01.

After the antitumor efficacy and tumor-targeting ability of the NP-FAP-DOX had been confirmed, its capacity to target CAFs and modulate TME composition was assessed. First, global Fap-α expression within tumors was analyzed by RT-qPCR, revealing a significant reduction following NP-FAP-DOX treatment compared with PBS and NP-CTR-DOX groups (Fig. 5A). Given that FAP-α can be expressed by multiple cell populations within the tumor, we next specifically investigated CAF-associated markers, due to their well-established role in tumor aggressiveness and therapy resistance. For this purpose, α-SMA immunofluorescence staining was performed, demonstrating a marked reduction in α-SMA-positive CAFs in NP-FAP-DOX-treated tumors (Fig. 5B).

To further evaluate the functional consequences of CAF depletion on ECM organization, CAF depletion was indirectly corroborated through Masson’s trichrome staining, which revealed reduced collagen content in the ECM of NP-FAP-DOX-treated tumors compared to control groups (Fig. 5C). SHG imaging further confirmed reduced collagen density and disrupted fiber alignment in NP-FAP-DOX-treated tumors, providing additional evidence of TME remodeling (Fig. 5D). These results indicate that NP-FAP-DOX efficiently targeted FAP-α-expressing tumor and stromal cells, reduced CAF abundance, promoted collagen degradation, and contributed to ECM homeostasis, thereby enhancing the antitumor therapy efficacy.

Given the well-established role of CAFs and ECM organization in regulating immune cell trafficking and function within tumors, we next investigated whether the stromal remodeling induced by NP-FAP-DOX translated into changes in tumor immune composition. A comprehensive flow cytometry panel was developed to analyze T lymphocytes (CD45^+^/CD3ε^+^), cytotoxic T cells (CD45^+^/CD3ε^+^/CD8a^+^), M1-like macrophages (CD45^+^/F4/80^+^/CD86^+^), and M2-like macrophages (CD45^+^/F4/80^+^/CD206^+^) (Fig. S8). NP-FAP-DOX treatment resulted in a marked increase in tumor lymphocyte infiltration, with a significant increase in the percentage of T lymphocytes (1.8-fold) and, more precisely, the cytotoxic T cells (1.7-fold) compared to the PBS group (Fig. 5E and F). In contrast, no changes in T cell infiltration were observed in the NP-CTR-DOX group. Regarding tumor-associated macrophages (TAMs), while no changes were observed in the percentage of M1-like macrophages (Fig. 5G), NP-FAP-DOX significantly decreased M2-like macrophage infiltration by approximately 27% compared to control tumors (Fig. 5H). This shift resulted in a 1.5-fold increase in the M1/M2 ratio (Fig. 5I), indicating a reversal of the immunosuppressive TME. In contrast, NP-CTR-DOX treatment did not alter TAM profiles.

Altogether, these results demonstrate that NP-FAP-DOX not only remodels the TME by targeting CAFs but also reverses the immunosuppressive environment of tumors, fostering immune activation and enhancing therapeutic potential.

### NP-FAP-DOX in vivo biocompatibility and safety

**Fig. 6.**
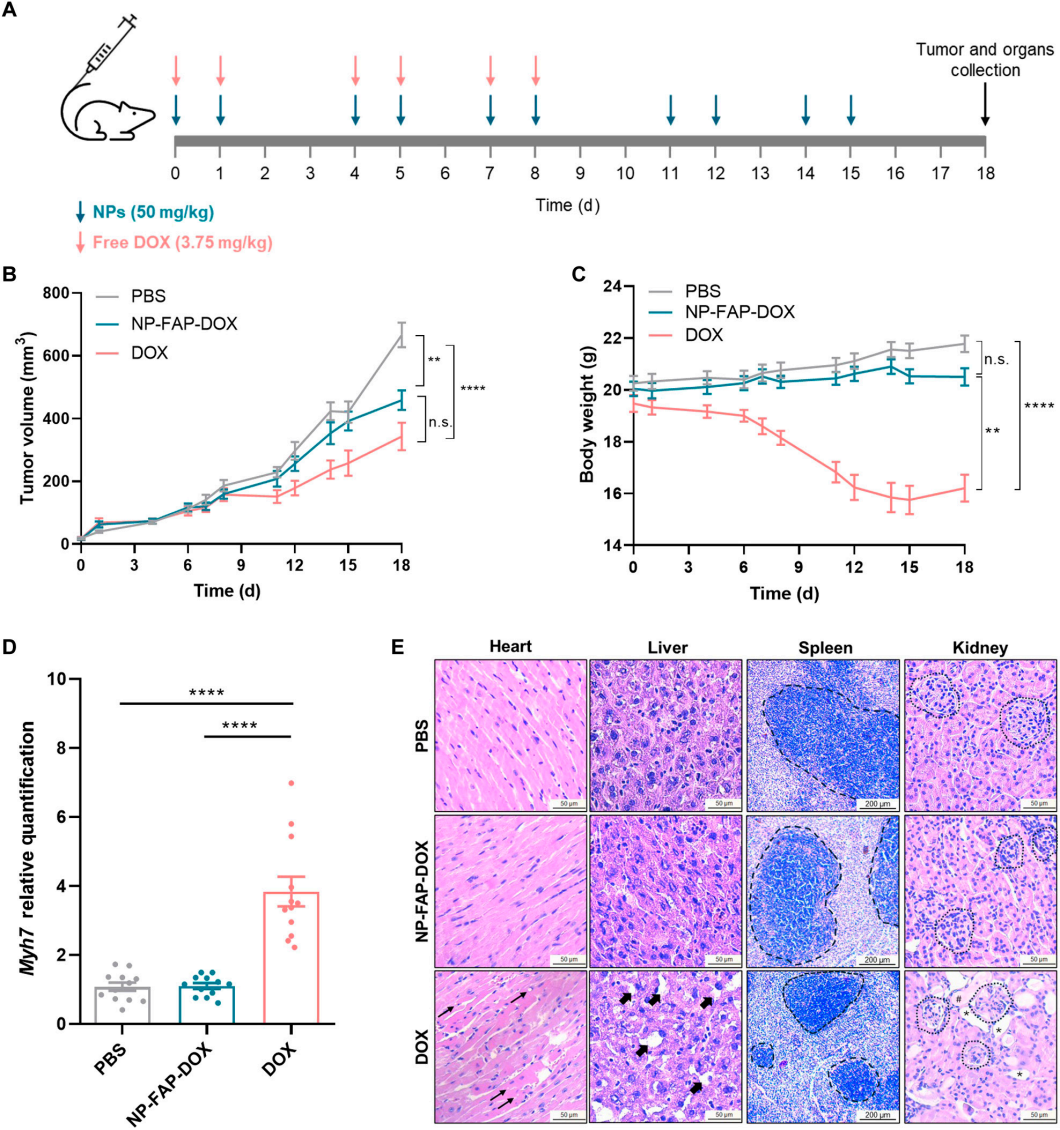
NP-FAP-DOX cardio and systemic toxicity evaluation. (A) Schematic representation of the in vivo study. 4T1 cells were injected into the mammary fat pad of BALB/C mice. The tumors, with an initial size of approximately 50 mm^3^, were intravenously treated with PBS (vehicle) or 50 mg/kg of nanoparticles (NPs: NP-CTR-DOX or NP-FAP-DOX) 4 times per week for 15 d (*n* = 12 per group). Free doxorubicin (DOX) (3.75 mg/kg) was intravenously administered for 8 d. The mice were sacrificed 3 d after the last treatment, and the tumor and organs were collected. (B) Tumor growth curve mice treated with PBS (vehicle), NP-FAP-DOX, and free DOX (*n* = 12 per group; mean ± SEM) (C) Body weight changes in tumor-bearing mice during treatment with PBS (vehicle), NP-FAP-DOX, or free DOX (mean ± SEM). (D) *Myh7* expression in heart tissue at the treatment endpoint was determined by RT-qPCR and normalized to PBS (mean ± SEM). (E) Representative images of hematoxylin and eosin staining in paraffin-embedded heart, liver, spleen, and kidney tissue sections at the treatment endpoint. Thin arrows indicate cytoplasmic lysosomes. Thick arrows indicate degeneration of hepatocytes. Dashed areas in the spleen represent the white pulp. Dotted areas in the kidney represent the glomerular capsule, the asterisk represents tubule dilations, and the hash sign represents cellular infiltrations. Scale bar: 50 μm (heart, liver, and kidney) or 200 μm (spleen). DOX: Free doxorubicin. ****P < 0.0001.

The safety profile of NP-FAP-DOX was compared to that of free doxorubicin in mice. Mice treated with an equivalent dose of free doxorubicin (3.75 mg/kg) showed severe toxicity, leading to the cessation of treatment after 8 d (Fig. 6A). In terms of antitumor efficacy, free doxorubicin treatment led to a significant reduction in tumor volume compared with PBS-treated animals (Fig. 6B). Tumor volumes in the free doxorubicin group remained numerically lower than those observed in NP-FAP-DOX-treated mice throughout the study; however, no statistically significant differences were detected between free doxorubicin and NP-FAP-DOX at the experimental endpoint.

Notably, the antitumor effect of free doxorubicin was achieved at the expense of pronounced systemic toxicity, as evidenced by rapid decline in body weight, ruffled fur, and hunching at the early stage of treatment (Fig. 6C), thereby limiting its therapeutic applicability. In contrast, NP-FAP-DOX treatment was well tolerated, with no signs of acute toxicity or compromised animal wellness.

The protective effects of NP-FAP-DOX against doxorubicin-induced toxicity were further assessed. The results confirmed that, unlike free doxorubicin, NP-FAP-DOX treatment did not induce cardiotoxicity, as evidenced by the normal levels of the marker of cardiac *atrophy myosin heavy chain β* (*Mhc-β*, *Myh7*) (Fig. 6D) and the absence of cytoplasmic vacuoles in NP-FAP-DOX-treated mice heart tissues (Fig. 6E). Moreover, hepatic, splenic, and renal toxicity were also evaluated. Free doxorubicin-treated mice showed severe tissue damage, including degeneration of hepatocytes, reduction in splenic white pulp, and structural deterioration in the kidney, such as aberrant Bowman’s space, tubule dilations, and cellular infiltrations (Fig. 6E). In contrast, these pathological changes were absent in NP-FAP-DOX-treated mice and the PBS-treated control group. Additionally, morphological analysis of the liver and spleen revealed significant atrophy in free doxorubicin-treated mice, whereas NP-FAP-DOX-treated mice exhibited no organ degeneration, maintaining normal tissue architecture (Fig. S9). Altogether, these results demonstrate that NP-FAP-DOX offers a safer alternative to free doxorubicin, effectively mitigating systemic toxicity while maintaining its therapeutic potential. The results underscore the applicability of NP-FAP-DOX as a tumor-specific nanodevice that minimizes adverse effects.

## Discussion

Several studies have demonstrated the pivotal role of CAFs in BC growth, metastasis, therapy resistance, and immunosuppression. This has motivated the development of CAF-targeted therapeutic strategies, some of which are currently under investigation in clinical trials for BC [31]. In this study, we developed NP-FAP-DOX, a CAF-targeted nanodevice designed to target CAFs as a therapeutic strategy for TNBC treatment. To address multiple TME-driven resistance mechanisms in TNBC, FAP-α, a surface marker selectively overexpressed in CAFs and associated with tumor progression, metastasis, and immune suppression in TNBC, was chosen as the target [14–16]. Additionally, recent studies have also reported elevated FAP-α expression in tumor cells and TAMs [32,33], further emphasizing its role as a therapeutic target within the TNBC microenvironment.

The NP-FAP-DOX’s characterization showed a hydrodynamic size of 247 ± 9 nm, which allows these NPs suitable for both passive and active mechanisms. The passive targeting mechanism is attributed to the enhanced permeability and retention effect in the tumor areas, allowing small-sized NPs to extravasate from the bloodstream through the tumors’ defective vasculature and accumulate in TME [34]. The combination of passive targeting with peptide functionalization enhances the ability of NP-FAP-DOX to specifically target FAP-α-expressing cells within the TME. Additionally, in vitro cargo release studies confirmed controlled doxorubicin delivery only in the presence of GSH, which is abundant intracellularly (up to 10 mM in cancer cells) [30]. GSH reduces dithiol bonds that anchor PEG to the NP surface, resulting in controlled doxorubicin release. In vitro biocompatibility studies further demonstrated a nontoxic profile in blood. Together, these results suggest that the NP-FAP-DOX content remains inside the pores until reaching the tumor, thereby preventing nonspecific doxorubicin delivery and adverse side effects.

Functionally, NP-FAP-DOX demonstrated specific targeting of FAP-α in BC cells with FAP-α-positive expression, as well as in CAFs derived from TNBC tumors. Moreover, the cytotoxic effect of NP-FAP-DOX was also confirmed. Remarkably, the NP-FAP-DOX presented good penetration efficiency in PDOs, which replicate the intrinsic characteristics of the tumor of origin and have potential use as a preclinical model in drug development [35]. In this 3D model, the NP-FAP-DOX maintained its targeting capability and cytotoxic effects.

In vivo, NP-FAP-DOX demonstrated the ability to target, accumulate, and deliver doxorubicin within the tumor. The designed nanodevice efficiently inhibited tumor growth by reducing the proliferation of tumor cells and inducing apoptosis. Importantly, treatment resulted in CAF depletion and tumor collagen reduction, consistent with the role of CAFs in therapeutic resistance and ECM remodeling [10,36]. It is known that interactions between tumor cells and ECM components can restrict drug availability at the tumor or induce adhesion-mediated drug resistance [37]. In fact, the ECM composition was associated with BC patients' prognosis, with a high tumor–stroma ratio correlating with a higher relapse rate and poorer overall survival [38]. While previous studies have shown that CAF-targeted or ECM-modulating nanotherapies can enhance drug penetration [39,40], stromal depletion alone does not always translate into a direct antitumor effect [41]. In contrast, NP-FAP-DOX exhibited robust antitumor activity beyond stromal remodeling, highlighting the benefit of combining CAF targeting with localized chemotherapy. In this context, although CAF-targeted nanotherapeutic strategies have been previously reported, including FAP-α-responsive systems or ECM-modulating NPs delivering doxorubicin [42,43], the present study provides several advances. Unlike prior approaches that rely on enzymatic ECM degradation or CAF-triggered NP disassembly, NP-FAP-DOX integrates active CAF targeting with a highly stable MSN-based nanocarrier, enabling controlled drug delivery while maintaining systemic stability. Most previous studies have focused on isolated aspects of TME remodeling—such as CAF depletion or ECM degradation—without integrating a comprehensive analysis of stromal, structural, and immune consequences. In contrast, the present work provides a more integrated characterization of how FAP-targeted NPs reshape the TME. Furthermore, the therapeutic response was extensively characterized using advanced patient-derived models, including patient-derived CAFs and PDOs, which better recapitulate tumor–stroma interactions observed in patients.

**Fig. 7.**
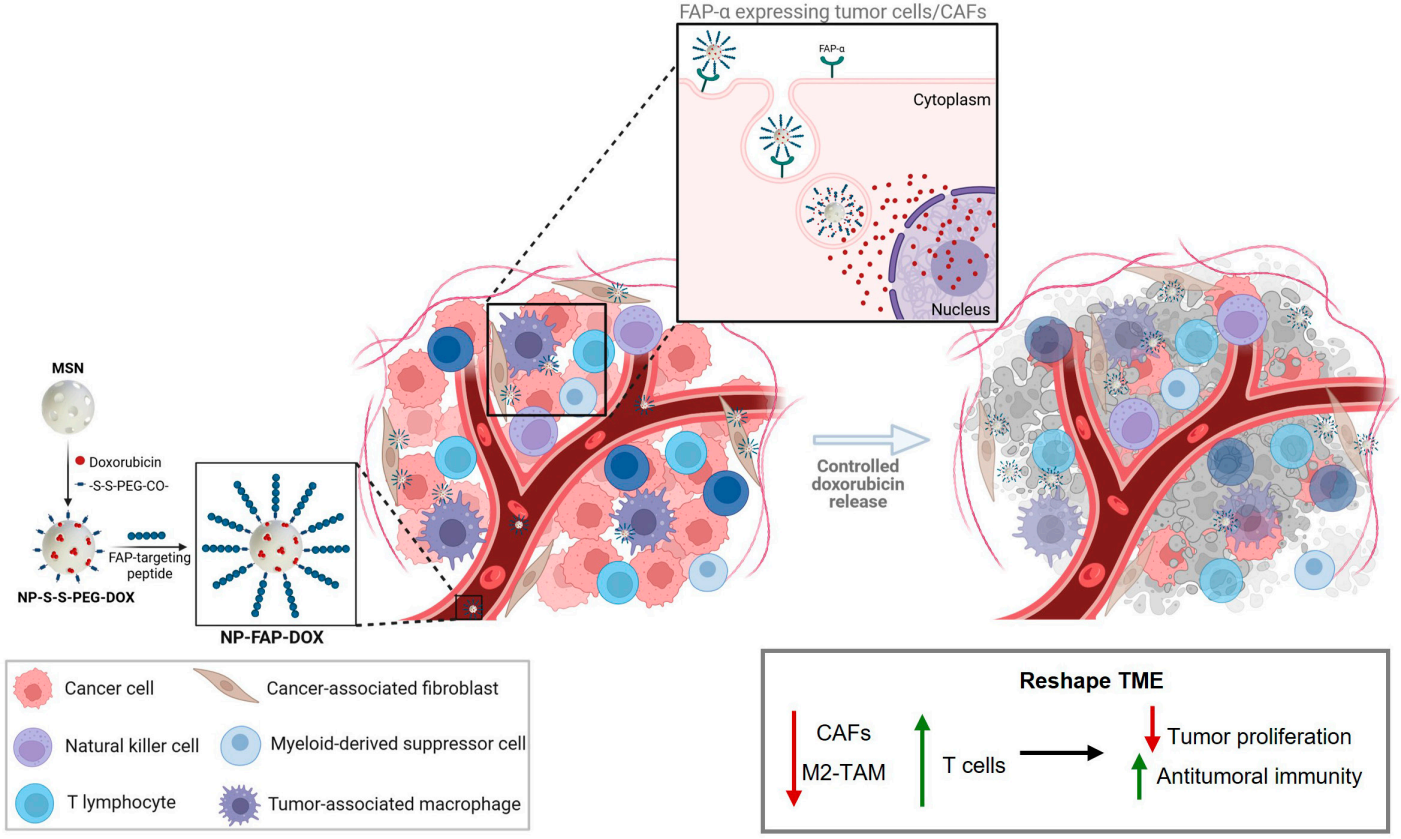
Schematic representation of the NP-FAP-DOX design and the proposed mechanism of action. MSNs were loaded with doxorubicin and functionalized with a FAP-α ligand peptide to enable selective recognition of FAP-α-expressing CAFs and tumor cells. Following cellular internalization, intracellular glutathione (GSH)-triggered cleavage of disulfide bonds induces controlled doxorubicin release. This results in CAF depletion, extracellular matrix degradation, induction of tumor cell apoptosis, and remodeling of tumor microenvironment, ultimately leading to potent antitumor activity in TNBC.

CAFs also play a critical role in modulating the tumor immune landscape [11]. In BC tumors, FAP-α-positive CAFs have been associated with an immunosuppressive function by inhibiting T cell proliferation and reducing immunotherapeutic efficacy [15,16]. Indeed, CAF depletion by FAP-based vaccines has been linked to enhanced antitumor immune responses in BC models, characterized by infiltration of dendritic and cytotoxic T cells, and reduced levels of myeloid-derived suppressor cells, TAMs, and regulatory T cells [44,45]. Similarly, the use of NPs that target CAFs in BC models has been shown to increase T cell infiltration in BC models [46,47]. In our study, NP-FAP-DOX treatment led to TME remodulation and activation of tumor immune response. Specifically, this treatment promoted tumor lymphocyte infiltration by increasing the percentage of T lymphocytes and cytotoxic T cells. Moreover, CAFs play a significant role in differentiating monocytes into M2-like macrophages in BC [48,49]. In TNBC, previous studies have demonstrated that the accumulation of M2-like macrophages is inversely correlated to the presence of cytotoxic T lymphocytes in the TME, leading to a poor prognosis [46]. Notably, in contrast with the previously reported NP studies, our nanodevice reduced the M2-like macrophages, thereby increasing the M1/M2 ratio. This effect may be partly attributed to FAP-α expression on TAMs, further supporting the multifunctional activity of NP-FAP-DOX [33]. CAFs and TAMs have been shown to act synergistically in colorectal cancer, contributing to progression and immune suppression [50]. Therefore, the dual stromal and immune-modulatory action of NP-FAP-DOX highlights its potential as a multifunctional nanotherapeutic strategy capable of overcoming TME-driven resistance mechanisms. To summarize the proposed mechanism underlying the therapeutic effects of NP-FAP-DOX within the tumor microenvironment, a schematic model is presented in Fig. 7.

Importantly, NP-FAP-DOX showed a clear improvement in the therapeutic and safety profile compared to free doxorubicin. While free doxorubicin induced significant tumor growth inhibition, this effect was achieved at a dose associated with severe systemic toxicity, including rapid decline in body weight, ruffled fur, and hunching at the early stage of treatment, leading to early treatment cessation. These findings highlight the narrow therapeutic window of free doxorubicin and underscore the advantage of NP-FAP-DOX in improving the therapeutic index by enabling sustained treatment, preserving animal welfare, and maintaining antitumor efficacy while minimizing adverse side effects.

Nevertheless, several limitations of our study should be acknowledged. First, although the orthotopic 4T1 murine model used here is immunocompetent and allows evaluation of immune modulation, it may not fully replicate the complexity and heterogeneity of human TNBC, particularly in terms of stromal and immune microenvironment diversity. Therefore, future studies should evaluate NP-FAP-DOX efficacy in more clinically relevant preclinical models, such as patient-derived xenografts (PDX), to better reflect human tumor biology. Second, while our findings support the potential of NP-FAP-DOX across FAP-α-positive tumors, further exploration of its efficacy in other BC subtypes is warranted. Finally, although the safety profile of NP-FAP-DOX was clearly superior to free doxorubicin in this study, comprehensive pharmacokinetic and long-term toxicity studies are necessary before clinical translation can be considered.

## Conclusion

In conclusion, our data highlight the potential of NP-FAP-DOX as a targeted drug delivery system for TNBC treatment. The nanodevice exhibited effective targeting of FAP-α, demonstrated cytotoxic activity in vitro, and showed significant antitumor efficacy in vivo. Additionally, NP-FAP-DOX modulated TME, reduced immune suppression, and improved safety by minimizing the side effects of free doxorubicin.

Overall, the NP-FAP-DOX represents a promising therapeutic strategy for TNBC by integrating localized chemotherapy and CAF depletion, promoting ECM homeostasis, and enhancing immune activation. Therefore, this work highlights the potential applicability of MSNs as therapeutic tools in the context of TNBC and other cancer types, although further studies are needed to translate these findings into clinical applications.

## Ethical Approval

BC PDOs and CAFs were obtained from TNBC tumor biopsies at the Biomedical Research Institute INCLIVA (Spain) (2014-VSC-PEA-0168) after ethical approval and signing of the informed consent. The in vivo experiments were approved by the Institutional Review Board of INCLIVA (2023/VSC/PEA/0287).

## Data Availability

All data generated and analyzed in this study are included in this article (and the Supplementary Materials). Any other additional information is available upon request.
