# Supplementary material for: Fibroblast Activation Protein Alpha-Targeted Nanoparticles for Tumor Microenvironment Remodeling and Antitumor Therapy in Triple-Negative Breast Cancer
Source: Biomater Res. 2026 Apr 8;30:0347. doi: 10.34133/bmr.0347 (PMC13058225; doi:10.34133/bmr.0347)
Supplement: Supplementary 1 — Figs. S1 to S9 Tables S1 to S6 [file bmr.0347.f1.docx]

**Fibroblast activation protein alpha-targeted nanoparticles for tumour microenvironment remodelling and antitumour therapy in triple-negative breast cancer**

Ana Lameirinhas^1,2^, Paula Díez^2,3,4^, Francisco J. Hicke^2,4^, Anna Ágreda-Roca^1^, Sandra Torres-Ruiz^1^, Paloma Sánchez-Serrano^1^, Marta Tapia^1,5^, Ana Lluch^1,5,6,7^, Begoña Bermejo^1,5,6,7^, Juan Miguel Cejalvo^1,5,6^, Ramón Martínez-Máñez^2,3,4,8, #^, Pilar Eroles^1,6,9, #^, Iris Garrido-Cano^1,2,4, #^

^1^ Biomedical Research Institute INCLIVA, Breast Cancer Biology Research Group, Valencia, Spain.

^2^ Instituto Interuniversitario de Investigación de Reconocimiento Molecular y Desarrollo Tecnológico (IDM), Universitat Politècnica de València, Universitat de València, València, Spain.

^3^ Unidad Mixta de Investigación en Nanomedicina y Sensores. Universitat Politècnica de València, Instituto de Investigación Sanitaria La Fe (IIS La Fe), València, Spain.

^4^ CIBER de Bioingeniería, Biomateriales y Nanomedicina (CIBER-BBN), Instituto de Salud Carlos III, Valencia, Spain.

^5^ Hospital Clínico Universitario de Valencia, Clinical Oncology Department, Valencia, Spain.

^6^ CIBER de Oncología (CIBERONC), Instituto de Salud Carlos III, Valencia, Spain.

^7^ University of Valencia, Department of Medicine, Valencia, Spain.

^8^ Unidad Mixta UPV-CIPF de Investigación en Mecanismos de Enfermedades y Nanomedicina, Universitat Politècnica de València, Centro de Investigación Príncipe Felipe, Valencia, Spain.

^9^ University of Valencia, Department of Physiology, Valencia, Spain.

^#^Corresponding authors: RMM: rmaez@qim.upv.es; PE: pilar.eroles@uv.es; IGC: igarrido@incliva.es

**Supplementary Figures**


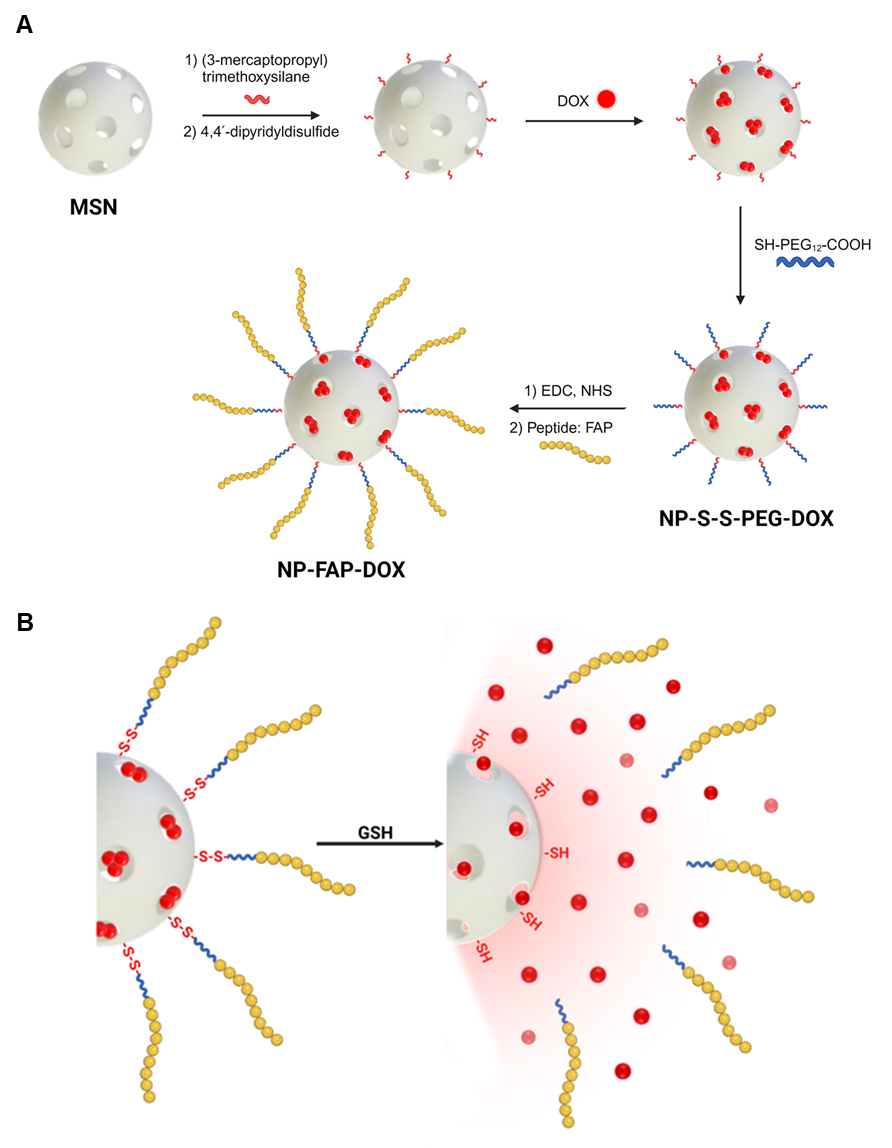


**Supplementary Figure S1. Schematic representation of NP-FAP-DOX (A)** Schematic synthesis procedure of NP-FAP-DOX. The pores of MSN were loaded with doxorubicin and their surfaces were coated with a redox-sensitive molecular gate formed by a disulphide bond attached to PEG and a peptide selective for FAP-α. **(B)** Schematic illustration of GSH-mediated DOX release of NP-FAP-DOX. The NP-FAP-DOX actively targets FAP-α-positive cells, and the molecular gate opens in the presence of GSH when the nanoparticles are internalized, ensuring controlled cargo release.

**
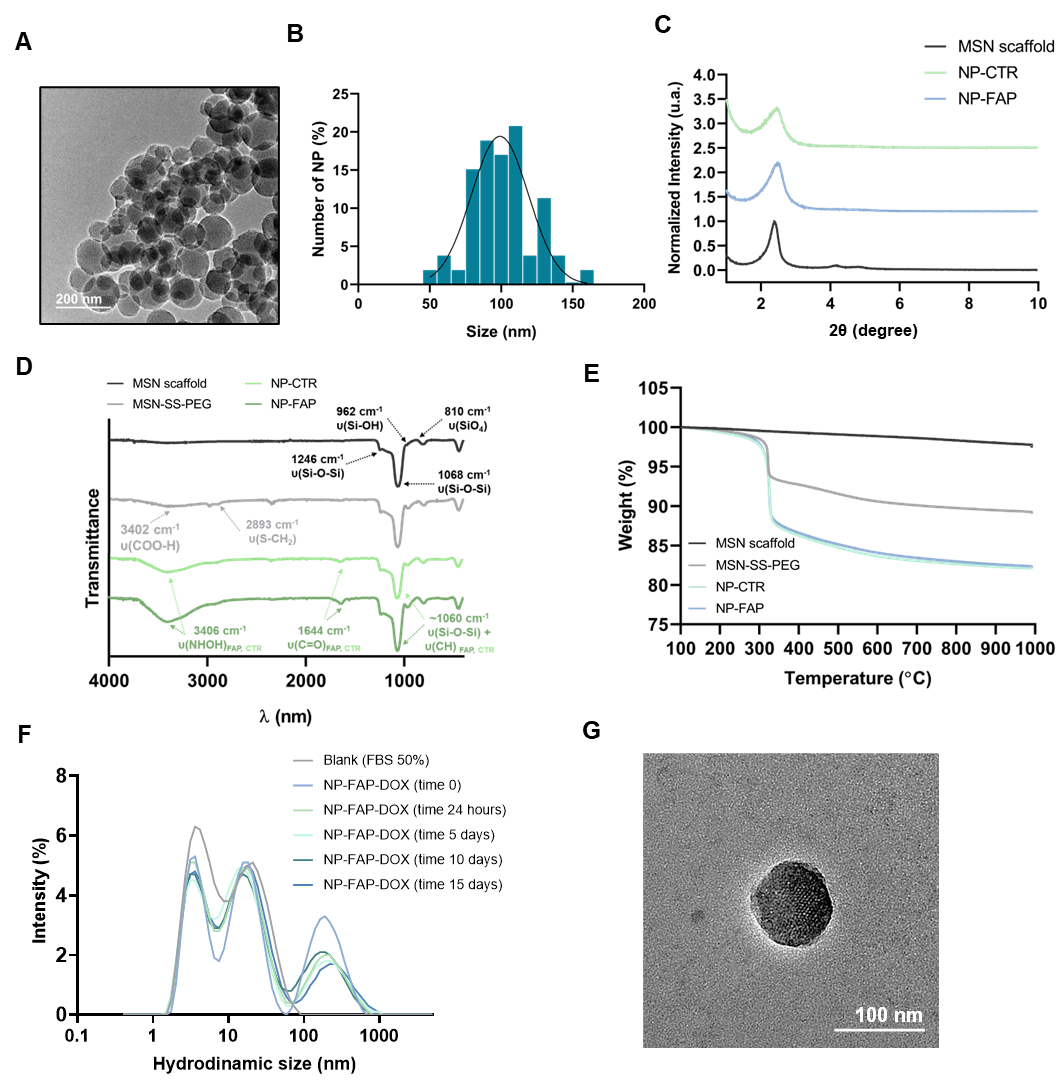
**

**Supplementary Figure S2. NP-FAP-DOX characterisation. (A)** TEM images of MSNs scaffold. Scale bar: 200 nm. **(B)** Histogram representing the size (diameter) distribution of MSNs determined by quantifying the dimensions of 50 NPs by TEM. **(C)** PDRX pattern at and low-angles of MSN scaffold, NP-CTR, and NP-FAP. **(D)** FTIR spectrum and **(E)** TGA of MSN scaffold, MSN-SS-PEG, NP-CTR, and NP-FAP. **(F)** Evaluation of NP-DOXO-FAP stability in 50% FBS. DLS intensity hydrodynamic diameter distribution for dispersions of NP-DOX-FAP incubated in 50% FBS at 37°C over time (0, 24 h, 5, 10, and 15 days).(**G**) TEM image of NP-FAP-DOX after 15 days of incubation in 50% FBS at 37 °C, showing preserved spherical morphology and intact mesoporous structure. Scale bar: 100 nm.


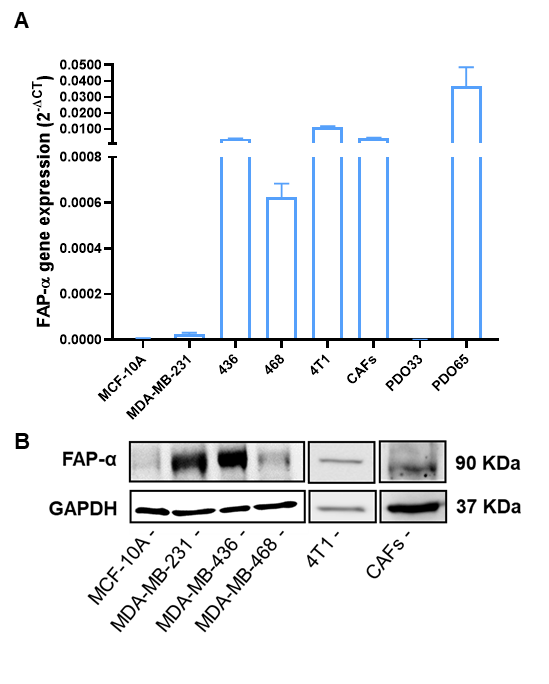


**Supplementary Figure S3. FAP-α expression.** Characterisation of FAP-α expression at mRNA and protein levels in the normal breast cell line MCF-10A, the TNBC cell lines MDA-MB-231, MDA-MB-436, MDA-MB-468, in murine TNBC cells 4T1, CAFs derived from TNBC biopsies, and in TNBC PDOs by RT-qPCR **(A)** and Western blot **(B)**(mean ± SD).


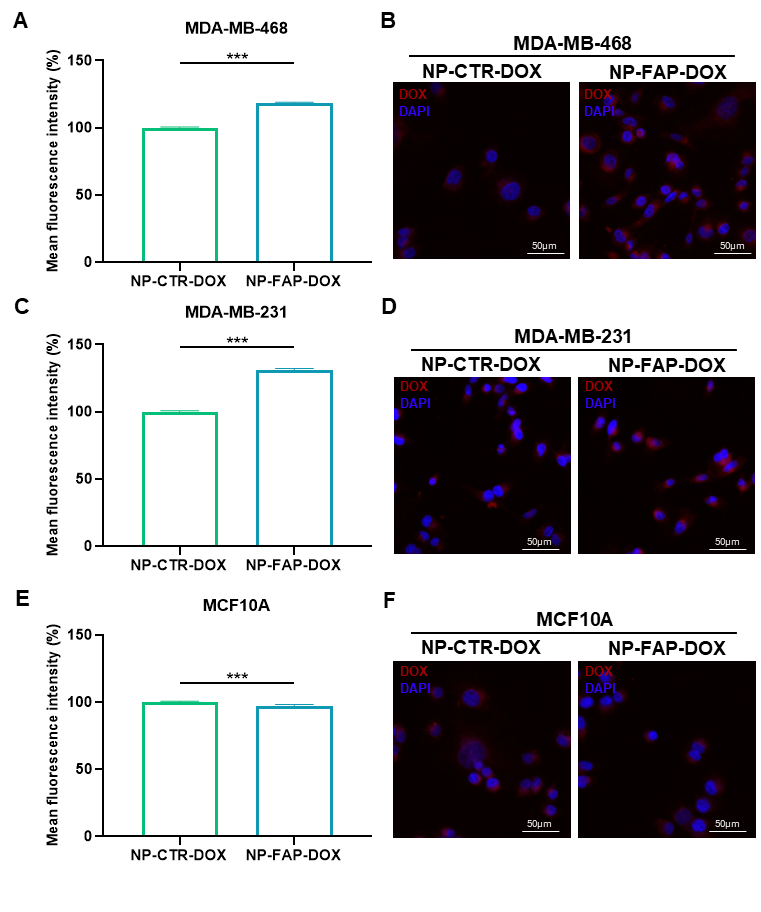


**Supplementary Figure S4. NP-FAP-DOX targeting efficacy**. Comparative Internalisation of NP-CTR-DOX vs. NP-FAP-DOX (25 µg/mL, 3 hours). Representative images and corresponding quantification of intracellular doxorubicin fluorescence in (A,B) MDA-MB-468, (C,D) MDA-MB-231, and (E,F) MCF10A cells. Fluorescence intensity normalised to NP-CTR-DOX (mean ± SEM). ***p < 0.001.

**
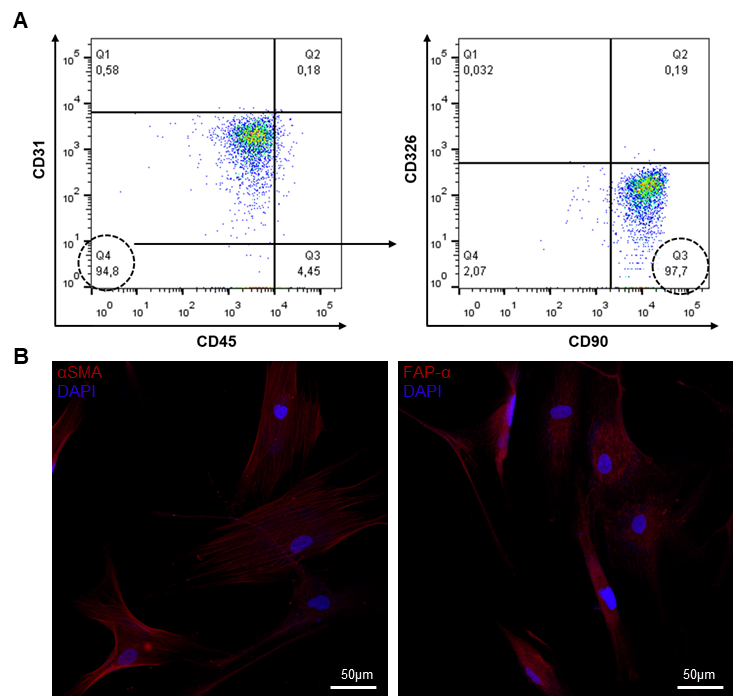
**

**Supplementary Figure S5. Characterisation of TNBC patient-derived CAFs.** (A) Representative flow cytometry gating strategy and marker expression confirming CAF phenotype (CD31⁻/CD45⁻/EpCAM⁻/CD90.1⁺). (B) Immunocytofluorescence images of CAFs showing positive staining for α-SMA (left) and FAP-α (right). Nuclei were counterstained with DAPI.


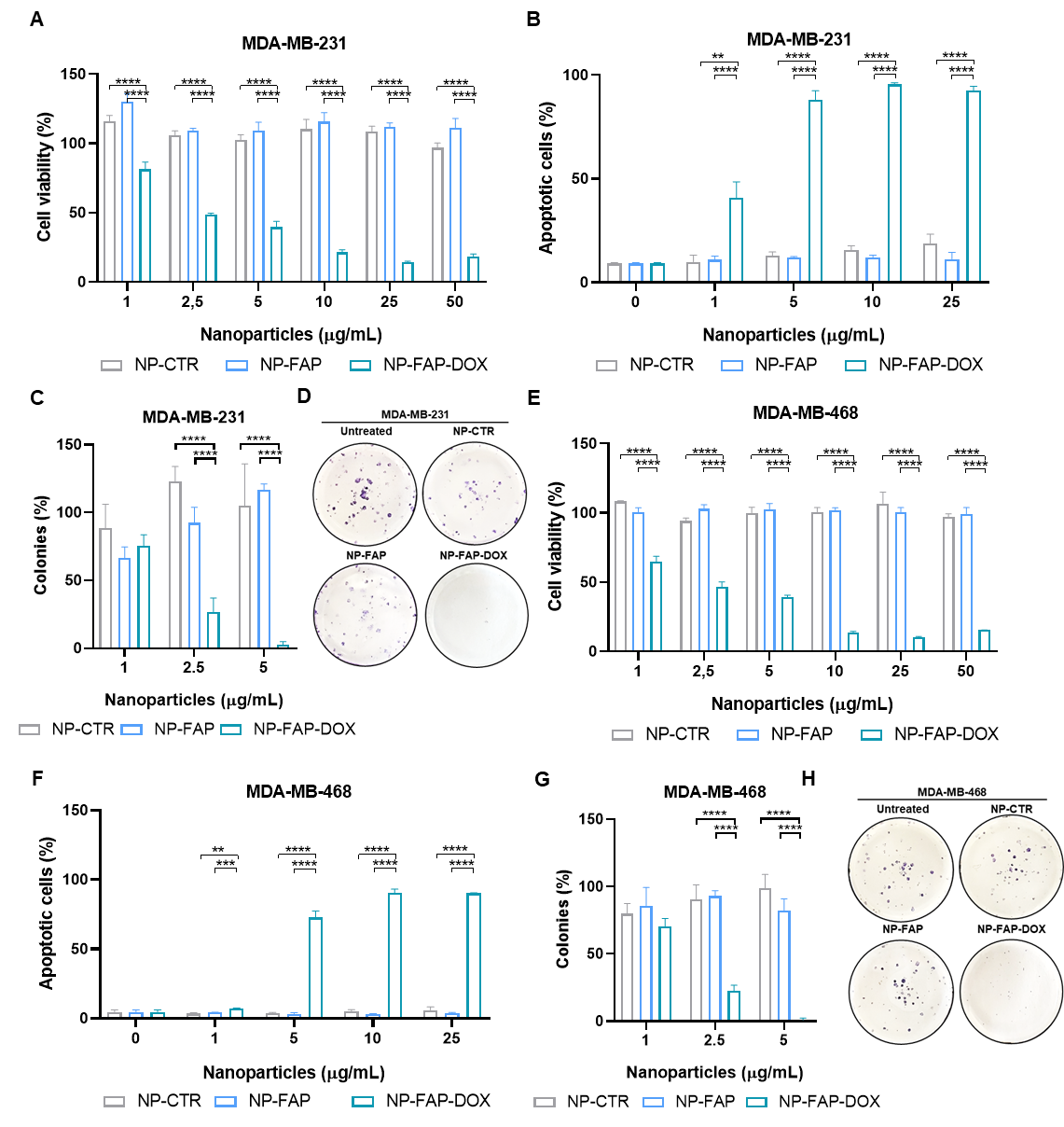


**Supplementary Figure S6. NP-FAP-DOX cytotoxic effect.** (**A–D**) MDA-MB-231, and (**E-H**) MDA-MB-468 cells were treated with NP-CTR, NP-FAP, or NP-FAP-DOX for 72 hours. Cell viability was measured using the WST-1 assay (**A, E**). Apoptosis was assessed by flow cytometry using Annexin V-FITC staining (**B, F**). Mean ± SD (n = 3). (**C-D, G-H**) Clonogenic assay in MDA-MB-231 (C-D) and MDA-MB-468 (G-H) cells treated with NP-CTR, NP-FAP, or NP-FAP-DOX (24 hours). Quantification of colony number normalised to the negative control (C, G). Representative images of colony formation after treatment with 5µg/mL (D, H). *p < 0.05, **p < 0.01, ***p < 0.001, ****p < 0.0001.


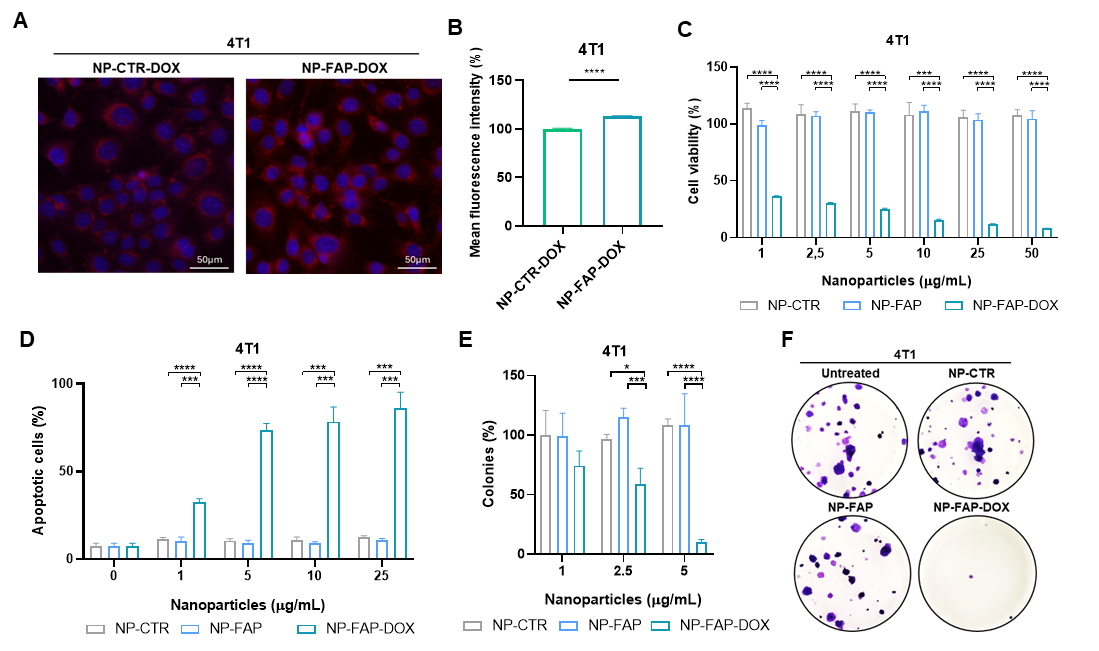


**Supplementary Figure S7. NP-FAP-DOX targeting efficacy and cytotoxic effect in 4T1 cells. (A-B)** Comparative Internalisation of NP-CTR-DOX vs. NP-FAP-DOX (25 µg/mL, 3 hours). Representative images (A) and corresponding quantification of intracellular doxorubicin fluorescence normalised to NP-CTR-DOX (mean ± SEM) (B). **(C-D)** Cells were treated with NP-CTR, NP-FAP, or NP-FAP-DOX for 72 hours, and cell viability was measured by WST-1 assay (C) and apoptosis was assessed by flow cytometry using Annexin V-FITC staining (D) (mean ± SD). (**E-F**) Clonogenic assay in 4T1 cells treated with NP-CTR, NP-FAP, or NP-FAP-DOX (24 hours). Quantification of colony number normalised to the negative control (E). Representative images of colony formation after treatment with 5µg/mL (F). *p < 0.05, ***p < 0.001, ****p < 0.0001.


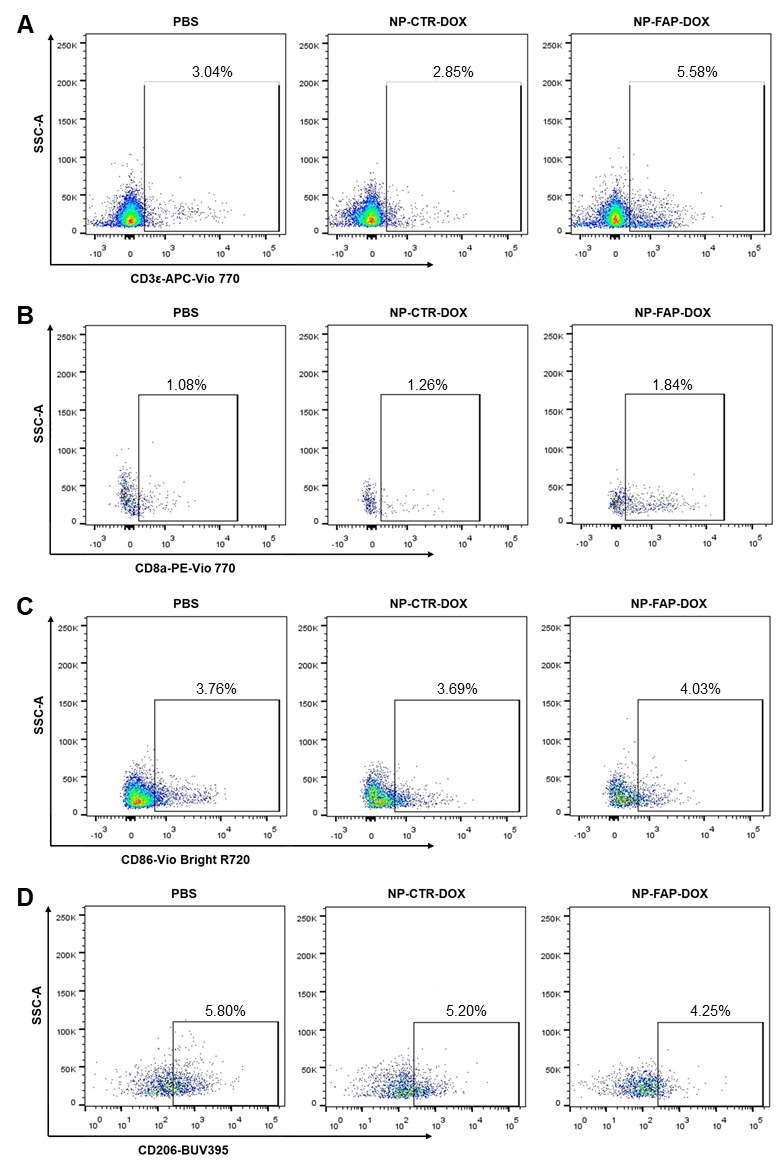


**Supplementary Figure S8. NP-FAP-DOX in vivo tumour microenvironment modulation.** Representative flow cytometry analysis of **(A)** T lymphocytes (CD45^+^/CD3ε^+^), **(B)** cytotoxic T cells (CD45^+^/CD3ε^+^/CD8a^+^), **(C)** M1-like macrophages (CD45^+^/F4/80^+^/CD86^+^), and **(D)** M2-like macrophages (CD45^+^/F4/80^+^/CD206^+^) in xenograft tumours analysed at the treatment endpoint.


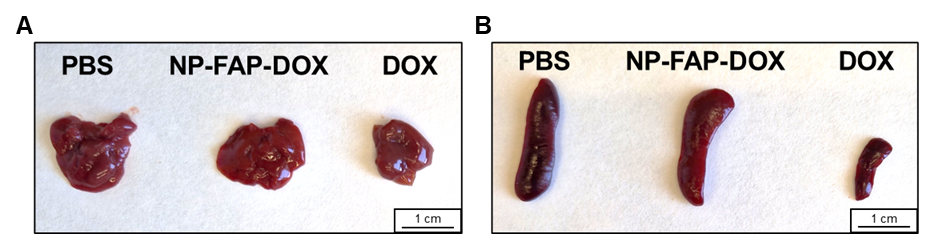


**Supplementary Figure S9.** **NP-FAP-DOX systemic toxicity assessment.** Representative **(A)** liver and **(B)** spleen morphology and size of tumour-bearing mice treated with PBS (vehicle), NP-FAP-DOX, or free DOX.

**Supplementary Tables**

**Supplementary Table S1.** Triple-negative breast cancer patient-derived organoids’ culture medium composition.

|  | Final concentration |
| --- | --- |
| Advanced-DMEM (Biowest) | N/A |
| Heat-inactivated fetal bovine serum (Gibco) | 5% (v/v) |
| L-glutamine (Biowest) | 1% (v/v) |
| HEPES (Gibco) | 10 μM |
| Gentamicin (Gibco) | 50 μg/mL |
| Human EGF (Merck) | 10 ng/mL |
| Y-27632 (Stemcell Technologies) | 10 μM |

**Supplementary Table S2.** Antibody panel for characterisation of immune cells in tumour microenvironment by flow cytometry.

| Flow cytometry panel | |
| --- | --- |
| Anti-CD326 (EpCAM), VioBlue^®^ | 130-117-759, Miltenyi Biotec |
| Anti-CD31, BV421 | 562939, BD Biosciences |
| Anti-CD45, FITC | 130-110-658, Miltenyi Biotec |
| Anti-CD3ε, APC-Vio^®^ 770 | 130-117-676, Miltenyi Biotec |
| Anti-CD8a, PE-Vio^®^ 770 | 130-118-946, Miltenyi Biotec |
| Anti-CD4, PE-Vio^®^ 615 | 130-118-316, Miltenyi Biotec |
| Anti-F4/80, PE | 130-116-499, Miltenyi Biotec |
| Anti-CD86, Vio^®^ Bright R720 | 130-128-565, Miltenyi Biotec |
| Anti-CD206, BUV395 | 568817, BD Biosciences |

**Supplementary Table S3.** Percentage in weight of organic content, SS-PEG, and peptide in nanoparticles obtained by thermogravimetry analysis.

| Nanoparticle | Total organic content  (μg mg^-1^ solid) | SS-PEG-COOH  (μg mg^-1^ solid) | Peptide  (μg mg^-1^ solid) |
| --- | --- | --- | --- |
| MSN scaffold | 262 | -- | -- |
| MSN-SS-PEG | 109.6 | 83.4 | -- |
| NP-CTR | 181.1 | 154.9 | 71.5 |
| NP-FAP | 178.3 | 152.1 | 68.7 |

**Supplementary Table S4.** Nanoparticles’ hydrodynamic size distribution obtained by dynamic light scattering measurements and polydispersity index (PDI).

| Nanoparticle | Size (nm) ± SD | PDI I ± SD |
| --- | --- | --- |
| MSN scaffold | 180 ± 2 | 0.242 ± 0.008 |
| NP-CTR | 276 ± 3 | 0.315 ± 0.026 |
| NP-FAP | 268 ± 3 | 0.400 ± 0.014 |
| NP-CTR-DOX | 246 ± 1 | 0.316 ± 0.010 |
| NP-FAP-DOX | 247 ± 9 | 0.369 ± 0.024 |

SD – standard deviation of 3 measurements

**Supplementary Table S5.** Nanoparticles’ ζ potential.

| Nanoparticle | ζ potential (mV) ± SD |
| --- | --- |
| MSN scaffold | -34.9 ± 0.404 |
| MSN-SS-PEG | -39.7 ± 0.808 |
| MSN-SS-PEG-DOX | -18.9 ± 1.970 |
| NP-CTR | -34.1 ± 0.709 |
| NP-FAP | -37.3 ± 0.513 |
| NP-CTR-DOX | -30.6 ± 0.586 |
| NP-FAP-DOX | -32.8 ± 0.794 |

SD – standard deviation of 3 measurements

**Supplementary Table S6.** Polydispersity index (PDI) of NP-FAP-DOX incubated in 50% FBS at 37°C over time (0 hours, 24 hours, 5, 10, and 15 days).

| Nanoparticle | PDI I ± SD |
| --- | --- |
| Blank (FBS 50%) | 0.420 ± 0.003 |
| NP-FAP-DOX (time 0) | 0.694 ± 0.008 |
| NP-FAP-DOX (time 24 hours) | 0.590 ± 0.005 |
| NP-FAP-DOX (time 5 days) | 0.606 ± 0.018 |
| NP-FAP-DOX (time 10 days) | 0.629 ± 0.005 |
| NP-FAP-DOX (time 15 days) | 0.595 ± 0.018 |
